# An Intelligent Magneto‐Mechanical Platform for Cellular Sensing in 3D Microenvironments

**DOI:** 10.1002/advs.202519132

**Published:** 2025-12-22

**Authors:** Yue Quan, Yuxin Wang, Sen Ding, Bingpu Zhou, Yinning Zhou

**Affiliations:** ^1^ Institute of Applied Physics and Materials Engineering Joint Key Laboratory of the Ministry of Education University of Macau Taipa Macau

**Keywords:** cell monitoring, cellular magneto‐mechanical sensing, closed‐loop monitoring, machine intelligence

## Abstract

Deciphering cellular proliferation mechanics in unperturbed 3D microenvironments remains challenging, as optical methods induce phototoxicity and fail in opaque matrices, while impedance sensing lacks spatial specificity and 3D compatibility. We introduce MagMI, a pioneering machine intelligence‐driven magneto‐mechanical sensing platform integrates arrays of magneto‐mechanical pillars to passively monitor nanoscale cellular force associated with proliferation in dense 3D cultures. Proliferation‐driven pillar deflections modulating magnetic fields are dynamically captured by high‐sensitivity Hall sensors. Across each experiment, MagMI acquires and processes over 1 × 10^6^ magneto‐mechanical data events, feeding bespoke machine‐learning models that serve as intelligent decoders—directly translating complex spatiotemporal magnetic signatures into quantitative maps portraying cellular dynamics without recourse to physical modeling. We validate MagMI by demonstrating its capabilities in: real‐time reconstruction and forecasting of proliferation kinetics at the population and single‐cell level; distinguishing multiple cell types via unique biomechanical phenotypes; and enabling fully closed‐loop experimentation via our integrated MagVizio suite for streaming analysis and automated feedback. MagMI is inherently label‐free, phototoxicity‐free, and compatible with optically opaque matrices. By delivering the first nanoscale force readout on tens of micrometer pillars, MagMI establishes a transformative approach for intelligent drug screening, systems mechanobiology, and broader investigations of cellular mechanics in physiologically relevant 3D settings.

## Graphical abstract

This study presents MagMI, a pioneering machine intelligence‐driven magneto‐mechanical sensing platform. It utilizes magneto‐mechanical arrays and machine learning to achieve label‐free, real‐time monitoring and classification of cellular proliferation dynamics within 3D microenvironments. MagMI establishes a transformative approach for intelligent drug screening, systems mechanobiology, and broader investigations of cellular mechanics in physiologically relevant 3D settings.

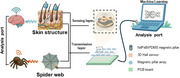

## 1 Introduction

Quantifying the dynamic behaviors of cells within 3D microenvironments over extended timescales is fundamental to understanding biological processes such as cancer invasion, drug resistance, and tissue morphogenesis [1, 2]. Advanced in vitro cancer cell screening platforms enable reduced drug development timelines and costs, coupled with improved reliability in predicting therapeutic efficacy and toxicological profiles [3, 4]. However, current methods for longitudinal, non‐invasive monitoring of cellular proliferation at single‐cell or micro‐colony resolution within physiologically relevant 3D architectures remain severely limited. Traditional endpoint assays (e.g., 3‐(4,5‐dimethylthiazol‐2‐yl)‐2,5‐diphenyltetrazolium bromide‐MTT, fluorescence staining, Cell Counting Kit‐8‐CCK‐8) disrupt the biological process and preclude continuous observation. For real‐time assessment, researchers have developed non‐invasive optical detection methods based on techniques such as Raman spectra [5, 6, 7] and terahertz spectroscopy [8, 9, 10]. Lee et al. utilized lens‐free shadow imaging technology (LSIT), based on digital inline holography (DIH), to monitor natural killer cell activity [11]. Caponi et al. assessed biochemical changes within cells based on variations in Raman band shapes [5]. However, these optical approaches face inherent limitations. High‐resolution optical tracking is not only computationally intensive but also incompatible with dense cell cultures or opaque growth media. Furthermore, the integration of such optics with microfluidic platforms introduces additional technical challenges—including alignment drift and refractive index mismatches—that compromise measurement accuracy. These limitations are compounded by phototoxic effects from prolonged laser exposure during time‐lapse imaging, which can significantly perturb normal cellular behavior and experimental outcomes.

Electric cell‐substrate impedance sensing (ECIS) has also been well developed as a dynamic, real‐time, non‐invasive, and label‐free modality for monitoring of cell growth and viability by exploiting the capacitance and resistance characteristics of cells adhering to microelectrodes [12, 13, 14, 15, 16, 17]. Advances such as Park et al.’s rapid capacitance‐based electrical drug monitoring system [18], the integration of IC (integrated circuit) with ECIS [19, 20], Chitale et al.’s high‐throughput impedance imaging in 96‐microplate formats [21], Hui et al.’s single‐cell pharmacodynamic evaluation [22] have significantly expanded its utility. However, ECIS remains fundamentally constrained by its electrode dimensions (generally >20 µm), which preclude subcellular force resolution and obscure micro‐colony heterogeneity. Moreover, impedance signals conflate multiple biophysical phenomena and cannot selectively attribute changes to proliferation‐specific mechanics, while measurements are inherently limited to planar surfaces and fail in physiologically relevant 3D matrices such as hydrogels or tumor spheroids.

The critical unmet challenge is the absence of an intelligent sensing platform capable of passively, continuously, and spatially resolving the subtle nanoscale biomechanical forces generated by proliferating cells within heterogeneous 3D populations, without perturbation. To address this critical gap, we introduce MagMI, an innovative machine intelligence‐driven magneto‐mechanical sensing platform that directly marries engineered soft matter with data‐centric inference, to intelligently decode cellular dynamics in a 3D microenvironment. Our approach leverages an array of soft, magnetically active micro‐pillars (composed of PDMS embedded with neodymium iron boron microparticles) that serve as programmable 3D mechanical scaffolds. The core premise of our MagMI platform is that cellular proliferation within a confined 3D microenvironment necessarily generates nanoscale expansive forces. We do not directly measure the biochemical process of proliferation itself (e.g., DNA replication). Specifically, the physical forces exerted by cells as they grow, divide, and push against the constraining magnetic micropillars. Crucially, as cells proliferate and exert nanoscale expansive forces within the pillar network, they induce microscale deflections in these pillars, modulating their intrinsic magnetic fields in a spatially encoded manner. The core innovation is a bespoke machine learning framework that learns the nonlinear mapping from high‐dimensional magnetic field perturbations to underlying proliferation kinetics across space and time, circumventing the need for explicit physical modeling or handcrafted features and enabling robust operation in dense, heterogeneous, and optically opaque cultures.

Successfully, we validate MagMI with (i) forecasting proliferation dynamics by converting real‐time magneto‐mechanical signals into continuous proliferation curves over extended culture periods in dense 3D cultures, (ii) classifying four distinct cell lines via their unique intrinsic biomechanical fingerprints by analyzing the force patterns generated during proliferation, and (iii) integrating these predictive and classificatory capabilities into a dedicated software suite to realize true closed‐loop intelligent monitoring. This software provides a user‐friendly interface for real‐time visualization of the magnetic field data, immediate prediction of proliferation dynamics, and automated cell type classification, facilitating continuous, intelligent assessment of cellular behavior within the 3D microenvironment. Thus, MagMI achieves not only real‐time monitoring of cell behavior but also provides an intelligent, interpretable platform for quantifying proliferation mechanics and cellular heterogeneity in physiologically relevant 3D settings, paving the way for advanced drug screening and personalized medicine applications.

## 2 Results

### 2.1 Device Design and Working Principle

**FIGURE 1:**
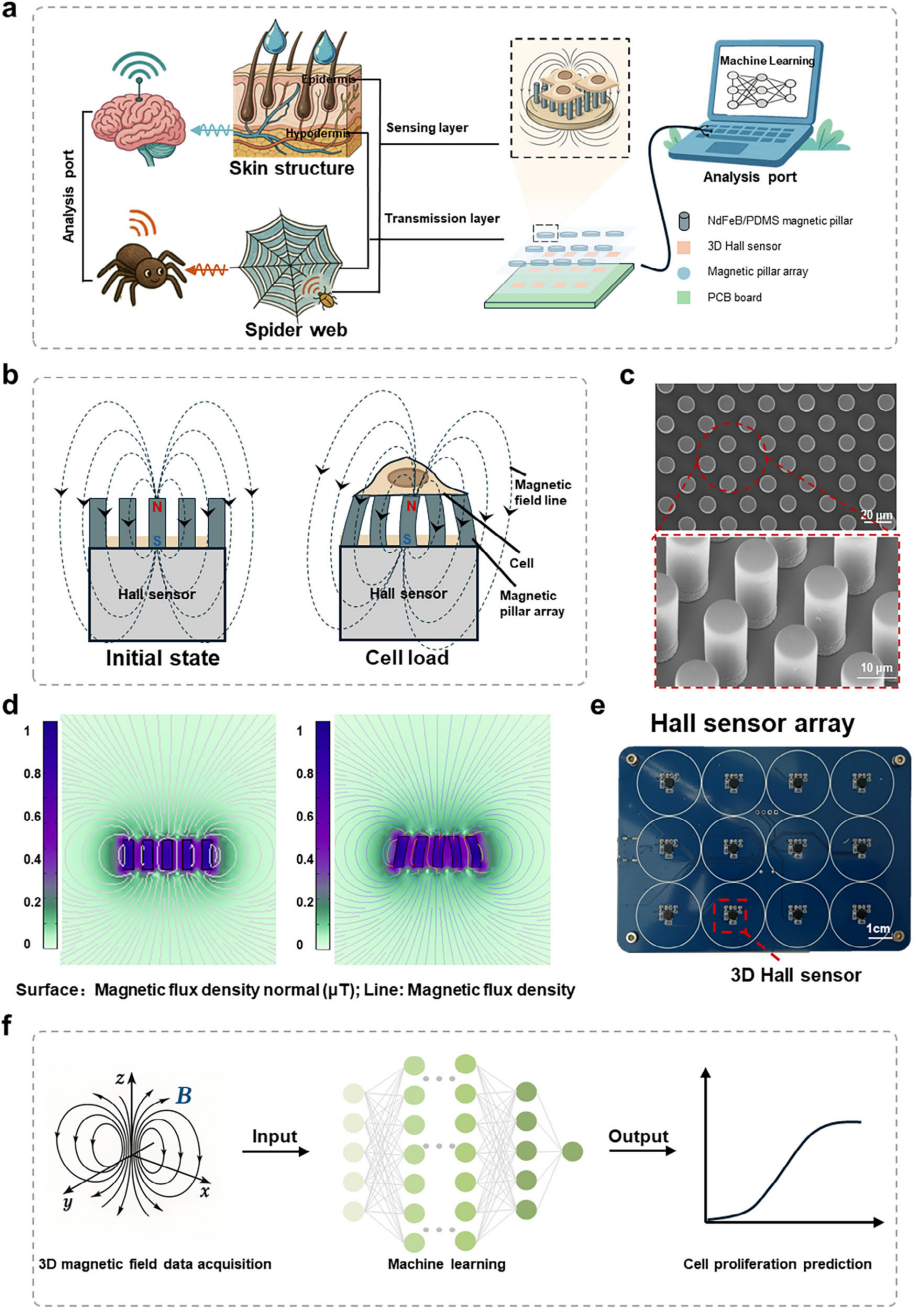
Structure design and working mechanism of the machine intelligence‐driven magneto‐mechanical sensing platform (MagMI). (a) Illustration of human skin sensing fluid flow, spider appendages detecting objects, and the MagMI detecting cell behavior. (b) Illustration of the architecture and working principle of the sensor. (c) SEM images of the magnetic micropillar arrays. (d) Simulation results of the magnetic field distribution around the magnetic film with micropillar arrays, and when the micropillar arrays have deflection. (e) Photograph of the Hall sensor array. (f) Schematic of detecting cellular behavior via machine learning using sensor‐detected magnetic field signals.

Inspired by the mechanosensitive principles of human skin hair sensing fluid flow and spider appendages detecting objects, we designed a flexible magnetic micropillar array sensor for non‐invasive, 3D, real‐time detection of cellular behavior (Figure 1a). Figure 1b illustrates the fundamental architecture and working principle of the sensor, which comprises three core components: (i) **Sensing layer**: this functional layer consists of a flexible magnetic thin film patterned with a micropillar array (Elastic modulus E≈ 0.9 MPa, thickness = 200 µm; see Figure S1). (ii) **Transmission layer**: Positioned beneath the sensing layer, this layer incorporates a 4x3 array of triaxial Hall effect sensors (QMC5883L). The corresponding circuitry design is detailed in Figure S2. (iii) **Analysis port**: this interface facilitates data acquisition and subsequent computational processing.

As shown in Figure 1c, the micropillars possess a diameter of 10 µm, a height of 30 µm, and a spacing of 10 µm, resulting in an aspect ratio of 1:3. Biologically, the 10 µm diameter supports stable attachment of cell bodies (e.g., HeLa cells, 15–20 µm in diameter) while the 30 µm height provides 3D space for cell protrusions, matching the scale of filopodia (typically 100–400 nm in diameter and several to tens of micrometers in length [23]) that mediate cell‐matrix interactions. This aspect ratio ensures sufficient mechanical flexibility for measurable deflection under nanoscale cellular forces while maintaining structural integrity. Energy‐dispersive X‐ray spectroscopy (EDS) confirmed the material composition of the magnetic film (Figure S3), demonstrating the homogeneous dispersion of magnetic particles within the silicone matrix. The NdFeB microparticles, serving as a prototypical hard magnetic material, can be permanently magnetized and exhibit excellent biocompatibility (Figure S4). Finite element analysis (FEA) simulations (Figure 1d) predict that external forces inducing micropillar deflection will generate measurable alterations in the spatial distribution of the magnetic flux density vector. Crucially, the Hall sensor array is engineered for compatibility with standard 12‐well cell culture plates (Figures 1e; S5). A key advantage of this design is its non‐contact sensing configuration. This architecture entirely eliminates the issue of long‐term immersion of electronic components within liquid culture media, thereby enabling sustained longitudinal cell studies. For practical application, the magnetic film is fabricated into circular discs (diameter = 2.1 cm) and integrated into the culture wells (Figure S6). The operational mechanism of the flexible magnetic micropillar array sensor is depicted in Figure 1f. Cells cultured atop the micropillar array exert nanoscale forces during adhesion, contraction, migration, and proliferation. These forces induce microscale deflections in the underlying magnetic micropillars. Such deflections perturb the local magnetic field. The underlying Hall sensor array continuously monitors perturbations in all three spatial components of the 3D magnetic field(*B_x_*,*B_y_*,*B_z_*)in real time. The acquired spatiotemporal magnetic field data are transmitted to the analysis port. Here, bespoke machine learning algorithms decode the complex spatiotemporal magnetic signature patterns, ultimately enabling the reconstruction of the corresponding 3D cellular behavior dynamics.

### 2.2 Biocompatibility Assessment and Cellular Dynamics on Micropillar Arrays

**FIGURE 2:**
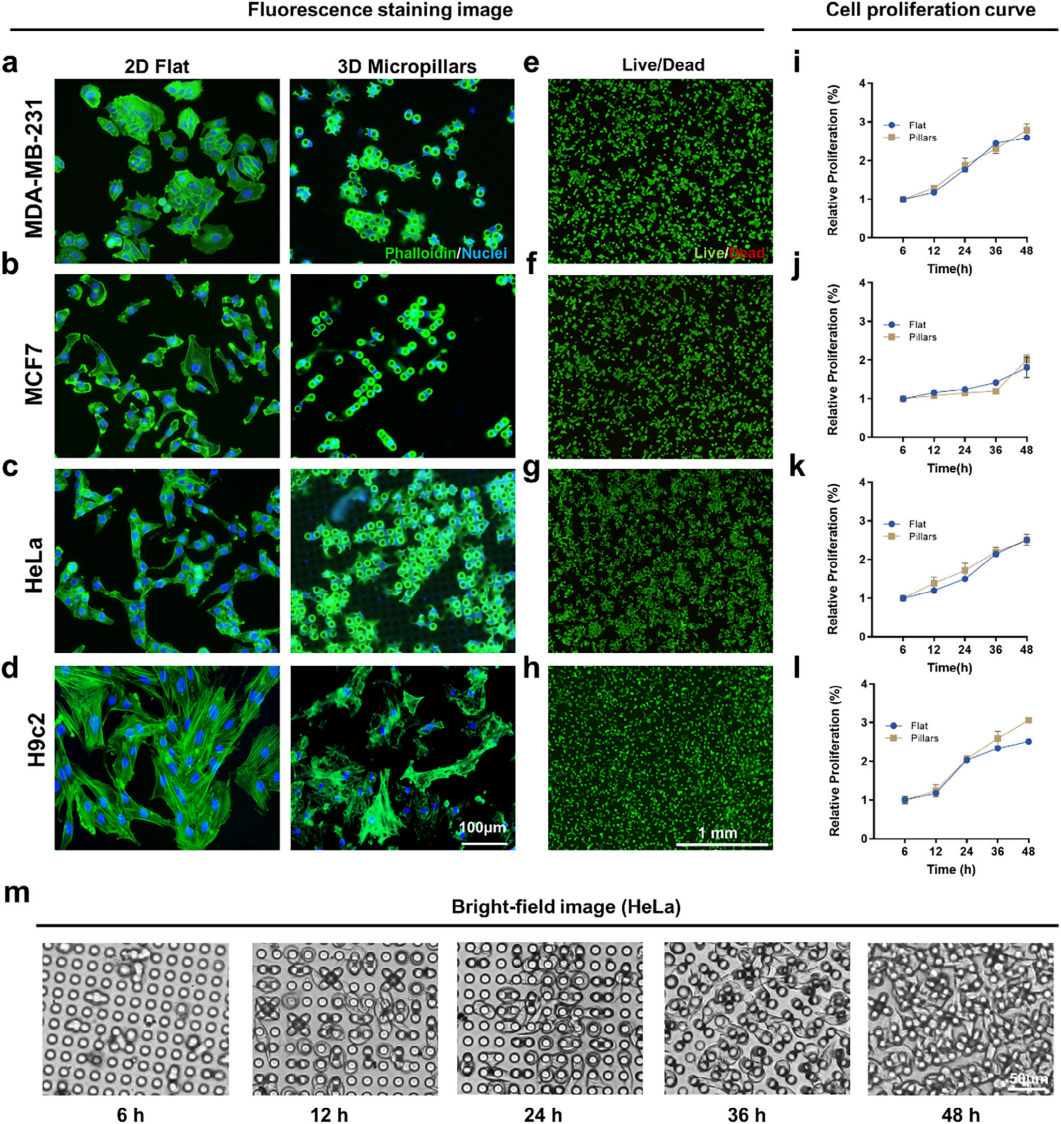
Characterization of cell growth on micropillar arrays. (a–d) Representative fluorescence images showing the (a) MDA‐MB‐231, (b) MCF7, (c) HeLa, and (d) H9c2 after 48 h of culture on flat and micropillar arrays. F‐actin (green) and cell nuclei (blue) were visualized through fluorescent staining. (e–h) After 48 h culture on micropillar array, the viability of (e) MDA‐MB‐231, (f) MCF7, (g) HeLa, and (h) H9c2 was determined by LIVE/DEAD kit, live (green), dead (red). (i–l) (i) MDA‐MB‐231, (j) MCF7, (k) HeLa and (l) H9c2 cell proliferation curve detected by CCK‐8 kit. Each group started with the same number of cells. (m) Bright‐field images of H9c2 morphology and number changes along with the time on the micropillar array.

To validate the biocompatibility and dynamic monitoring capacity of the MagMI platform, we systematically evaluated four cell types (MDA‐MB‐231, MCF7, HeLa, H9c2) cultured on micropillar arrays. Phalloidin‐based fluorescence imaging revealed uniformly distributed cytoskeletal morphology across all cell types (Figure 2a–d). Actin stress fibers exhibited diagonal orientation parallel to inter‐pillar gaps, with no evidence of membrane rupture or aberrant contraction. Compared to the flat group, cells on micropillar arrays demonstrated reduced cellular footprint area and increased height‐to‐width ratio, confirming a verticalized growth phenotype. Crucially, these topological adaptations occurred without compromising cellular fitness: Live/Dead assays verified >95% viability in all cell lines (Figure 2e–h) with minimal apoptosis and absence of necrosis. Proliferation kinetics over 48 h showed that the magnetic micropillar array did not inhibit cell proliferation compared with the flat group. Figure 2m shows the proliferation changes of HeLa over a period of 48 h (other cell lines are provided in Figure S7). Bright‐field imaging confirmed that micropillars physically segregated individual cells, reducing intercellular junctions and effectively suppressing collective behaviors. This autonomous spatial segregation of functionally intact cells establishes an ideal bio‐interface where topology‐enhanced single‐cell isolation synergizes with preserved physiology to enable noise‐suppressed magnetic signal acquisition.

### 2.3 Machine Learning‐Driven Decoding of 3D Magneto‐Mechanical Cell Proliferation Dynamics

To enable real‐time, label‐free decoding of cell proliferation within 3D microenvironments, we developed a magnetic‐field‐based sensing platform that intelligently couples magneto‐mechanical effect with machine learning. This integrated system reconstructs the dynamic 3D magnetic field distortions produced by cell‐induced deflections of a soft, magnetically responsive micropillars array, and translates these high‐dimensional spatiotemporal patterns into predictive models of growth behavior via data‐driven modeling.

**FIGURE 3:**
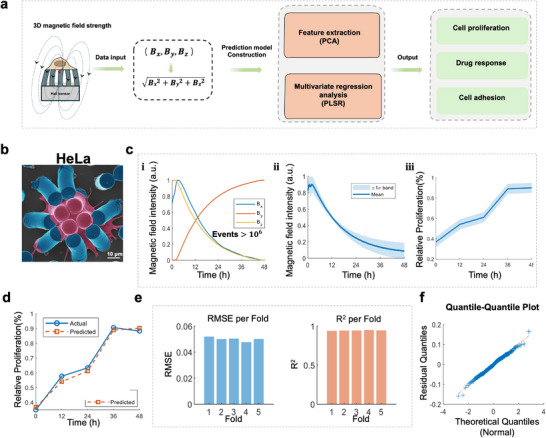
3D magnetic‐field modeling and cross‐validated prediction of HeLa cellular responses. (a) Schematic of 3D magnetic‐field computation showing the resultant (composite) field and the algorithmic workflow, including model inputs (magnetic‐field data) and predicted outputs. (b) SEM images HeLa cell growth around micropillars (scale bar = 10 µm). (c) (i, 3D magnetic‐field visualization; ii, time course of the resultant field magnitude (|B|); (iii) measured cellular absorbance (OD value) versus time. (d) machine learning‐predicted relative proliferation (OD value, normalized to the initial time point and expressed as Relative Proliferation) versus time. (e) fivefold cross‐validation metrics (per‐fold RMSE and R^2^). (f) Plus model evaluation: Quantile‐Quantile (Q‐Q) plot.

The end‐to‐end computational pipeline is illustrated in Figure 3a. Magnetic field data were continuously acquired, merged, and processed as inputs to supervised regression models that output time‐resolved predictions of cellular responses, including cell proliferation rate, drug sensitivity, and adhesion metrics. We validated the platform across four phenotypically distinct cell lines: HeLa, MCF7, MDA‐MB‐231, and H9c2. First, HeLa were observed. Their spatial expansion was carried out using scanning electron microscopy (SEM). The force exerted by the cells on the micropillars causes them to deflect. This mechanical deformation alters the magnetic field around the pillars, which is captured as time‐varying signals by the Hall sensors. As shown in Figure 3c, we (i) visualized the 3D magnetic fields topology, (ii) computed the temporal evolution of total field magnitude, and (iii) tracked corresponding cell proliferation via OD (optical density) measurements. These spatiotemporal magnetic signatures are then fed into a machine learning model trained to infer the proliferation kinetics. The machine learning‐predicted growth curves closely aligned with experimental data across all cell types (Figure 3d), with fivefold cross‐validation (Figure 3e) demonstrating high predictive consistency.

**FIGURE 4:**
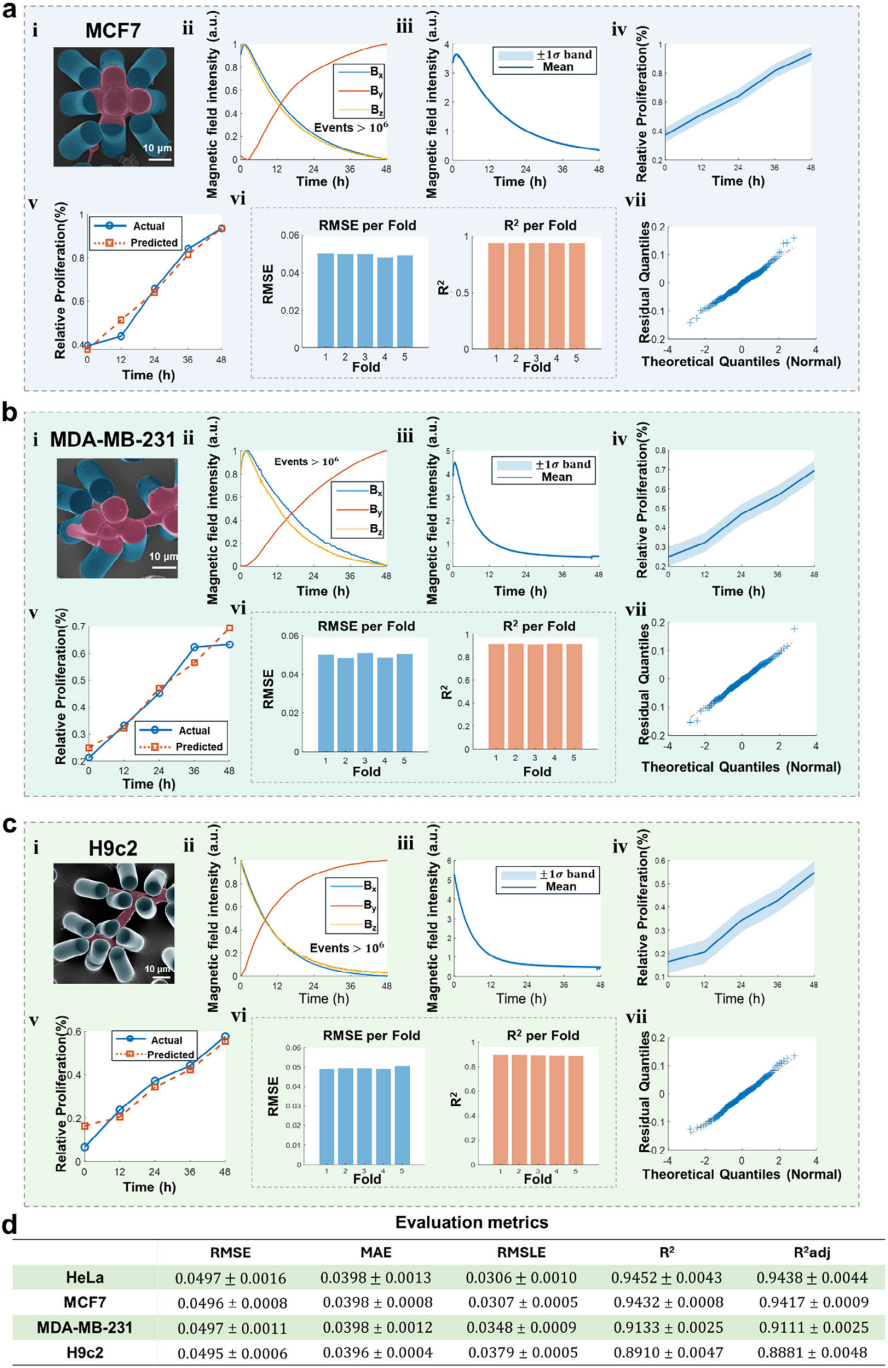
3D magnetic‐field modeling and cross‐validated prediction of MCF7, MDA‐MB‐231, and H9c2 cellular responses. For each cell line, (a) MCF7, (b) MDA‐MB‐231, and (c) H9c2: (i, SEM images cell growth around micropillars (scale bar = 10 µm); ii, 3D magnetic‐field visualization; iii, time course of the resultant field magnitude (|B|); iv, measured cellular relative proliferation (OD value) versus time; v, machine learning‐predicted relative proliferation (OD value, normalized to the initial time point and expressed as Relative Proliferation) versus time; vi, fivefold cross‐validation metrics (per‐fold RMSE and R^2^). vii, Plus model evaluation: Quantile‐Quantile (Q‐Q) plot. (d) Comparative performance matrix summarizing regression accuracy across the four cell lines (RMSE, MAE, RMSLE, R^2,^ and adjusted R^2^).

Critically, the machine learning framework demonstrated universal capability in decoding proliferation dynamics by establishing a direct mapping between time‐varying 3D magnetic field signatures and cellular relative proliferation. Predictions consistently matched empirical measurements with narrow confidence intervals and stable error distributions across validation folds. Quantile‐Quantile (Q‐Q) plots (Figure 3f) indicated near‐Gaussian residual distributions, confirming model calibration and the absence of systematic bias. Having established the workflow and its validation in HeLa cells, we applied the same MagMI platform to decode the proliferation dynamics of three additional cell lines: MCF7, MDA‐MB‐231, and H9c2 (Figure 4). For each cell line, the temporal evolution of the resultant magnetic field (|B|, Figure 4a‐ii,b‐ii,c‐ii) served as the key input. The machine learning models successfully predicted the proliferation curves (Figure 4a‐v,b‐v,c‐v), with the near‐Gaussian distribution of residuals in the Q‐Q plots (Figure 4a‐vii,b‐vii,c‐vii) indicating well‐calibrated models.

We further evaluated model performance using five standard regression metrics: root mean square error (RMSE), mean absolute error (MAE), root mean squared logarithmic error (RMSLE), coefficient of determination (R^2^), and adjusted R^2^. Cross‐model performance metrics (Figure 4d) confirmed strong generalization across cell types, with RMSE, MAE, RMSLE, R^2^ values remaining stable and aligned across validation folds. Importantly, adjusted R^2^ closely tracked raw R^2^, indicating minimal overfitting and reaffirming the models’ capacity to extract biologically relevant features from complex magnetic field data.

Altogether, these results highlight MagMI's capacity as a non‐invasive, optics‐free platform for real‐time decoding of 3D cell proliferation across diverse biological systems, with potential extensibility toward drug response and adhesion profiling. Its consistent performance across diverse cell types and microenvironments—coupled with scalability, compatibility with optically opaque systems, and applicability to ancillary endpoints like drug response—positions this machine intelligence‐integrated system as a transformative tool for intelligent in vitro modeling and automated biological discovery.

### 2.4 Machine Learning‐Based Decoding of Drug‐Modulated Proliferation via 3D Magnetic Signals

**FIGURE 5:**
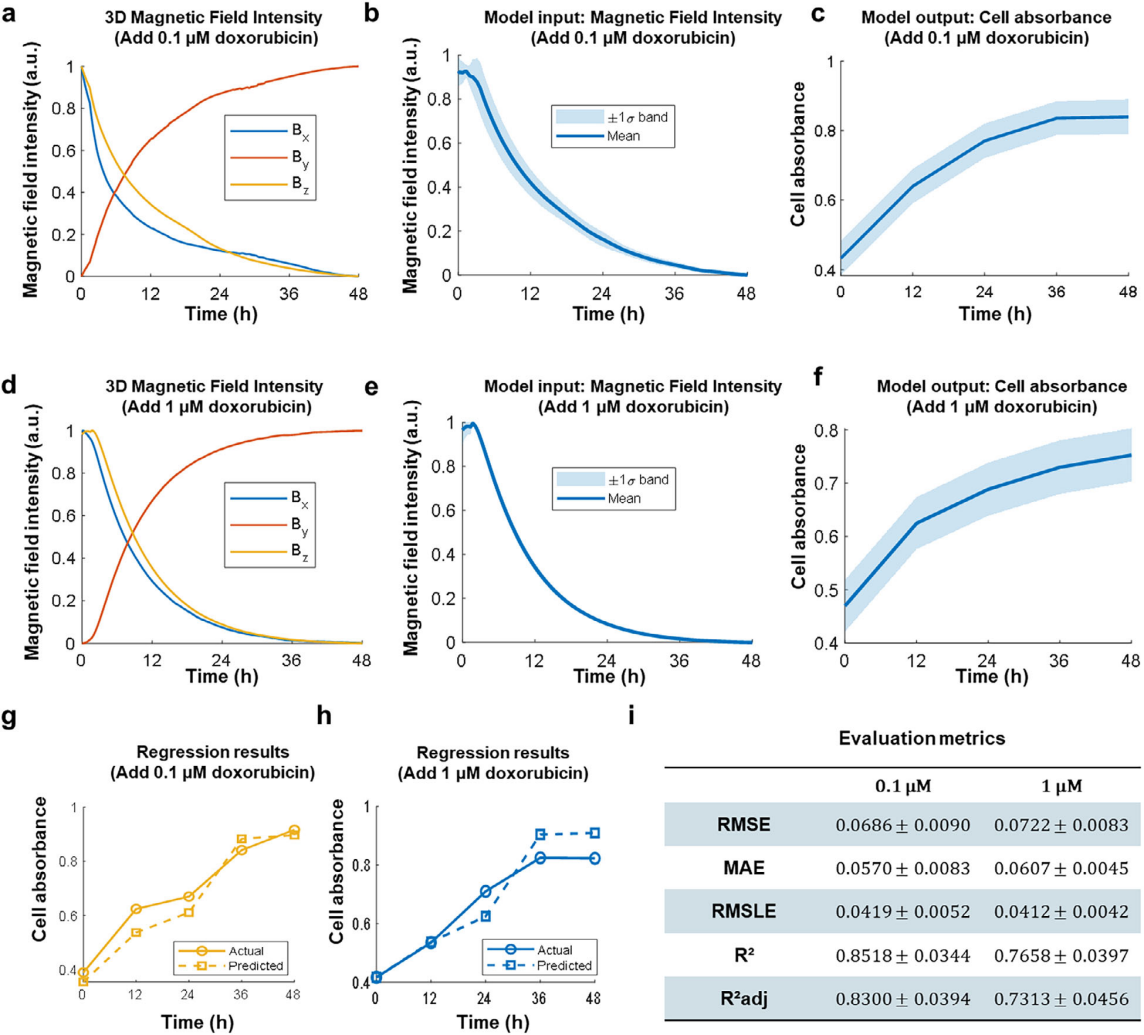
Machine learning‐based modeling of drug‐exposed HeLa cell proliferation. (a) Visualization of 3D magnetic field distribution following exposure to 0.1 µM doxorubicin. (b) Computed resultant magnetic field used as model input. (c) Time‐resolved cellular absorbance (OD value) used as model output. (d–f) Corresponding results for 1 µM doxorubicin: (d) 3D magnetic field visualization, (e) resultant field input, (f) absorbance output. (g,h) Representative regression results under 0.1 µM and 1 µM doxorubicin treatment, respectively. Machine learning‐predicted cellular absorbance (OD value, normalized to the initial time point and expressed as Relative Proliferation) versus time. (i) Comparative performance matrix summarizing regression accuracy in HeLa cells exposed to drug treatment, evaluated using RMSE, MAE, RMSLE, R^2^ and adjusted R^2^.

To assess the capacity of the MagMI in detecting drug‐induced alterations in cell proliferation, we trained regression models to decode the effects of anticancer drug doxorubicin (DOX) on HeLa cell growth dynamics based solely on 3D magnetic field perturbations. Following exposure to 0.1 and 1 µM doxorubicin, the magnetic field distributions around the micropillar arrays exhibited clear temporal decay patterns with component‐specific dynamics (Figure 5a,d). Under both conditions, the resultant magnetic field magnitude |B| showed monotonic decline over time (Figure 5b,e), consistent with reduced cell‐induced mechanical tension during cytotoxic progression. These magnetic signals served as model inputs, while the corresponding optical density (OD) trajectories, serving as a proxy for cell proliferation, were used as model outputs (Figure 5c,f).

Regression models trained on these magneto‐mechanical profiles successfully predicted OD evolution in both drug conditions, with strong alignment between predicted and measured curves (Figure 5g,h). Quantitatively, the model achieved high predictive accuracy across all regression metrics (Figure 5i): for 0.1 µM doxorubicin, RMSE  =  0.069 ± 0.009, MAE  =  0.057 ± 0.008, R^2^ = 0.85 ± 0.03; for 1 µM, RMSE = 0.072 ± 0.008, MAE = 0.060 ± 0.004, R^2 ^= 0.76 ± 0.04. The slight decrease in R^2^ and adjusted R^2^ at higher concentration suggests greater biological variability and morphological disintegration under cytotoxic stress, making the mechanical signature more heterogeneous and harder to decode. Notably, RMSLE values remained low (0.041–0.042), indicating robust performance even for small‐magnitude fluctuations, critical for early‐phase drug response detection. These results validate that the magnetic field decay induced by cellular biomechanical weakening under chemotherapy can be reliably captured and decoded via machine learning, offering a non‐optical, label‐free method for monitoring drug efficacy.

Together, these findings demonstrate that the MagMI platform sensitively detects drug‐induced proliferation suppression via mechano‐signature and captures dose‐dependent effects on biomechanical output. This positions the system as a powerful tool for real‐time pharmacodynamic profiling, enabling early discrimination of cytostatic vs. cytotoxic responses within physiologically relevant 3D microenvironments, without the need for invasive optical labeling or impedance electrodes.

### 2.5 Machine Learning‐Based Classification of Cell Phenotypes from Magnetic Field Features

**FIGURE 6:**
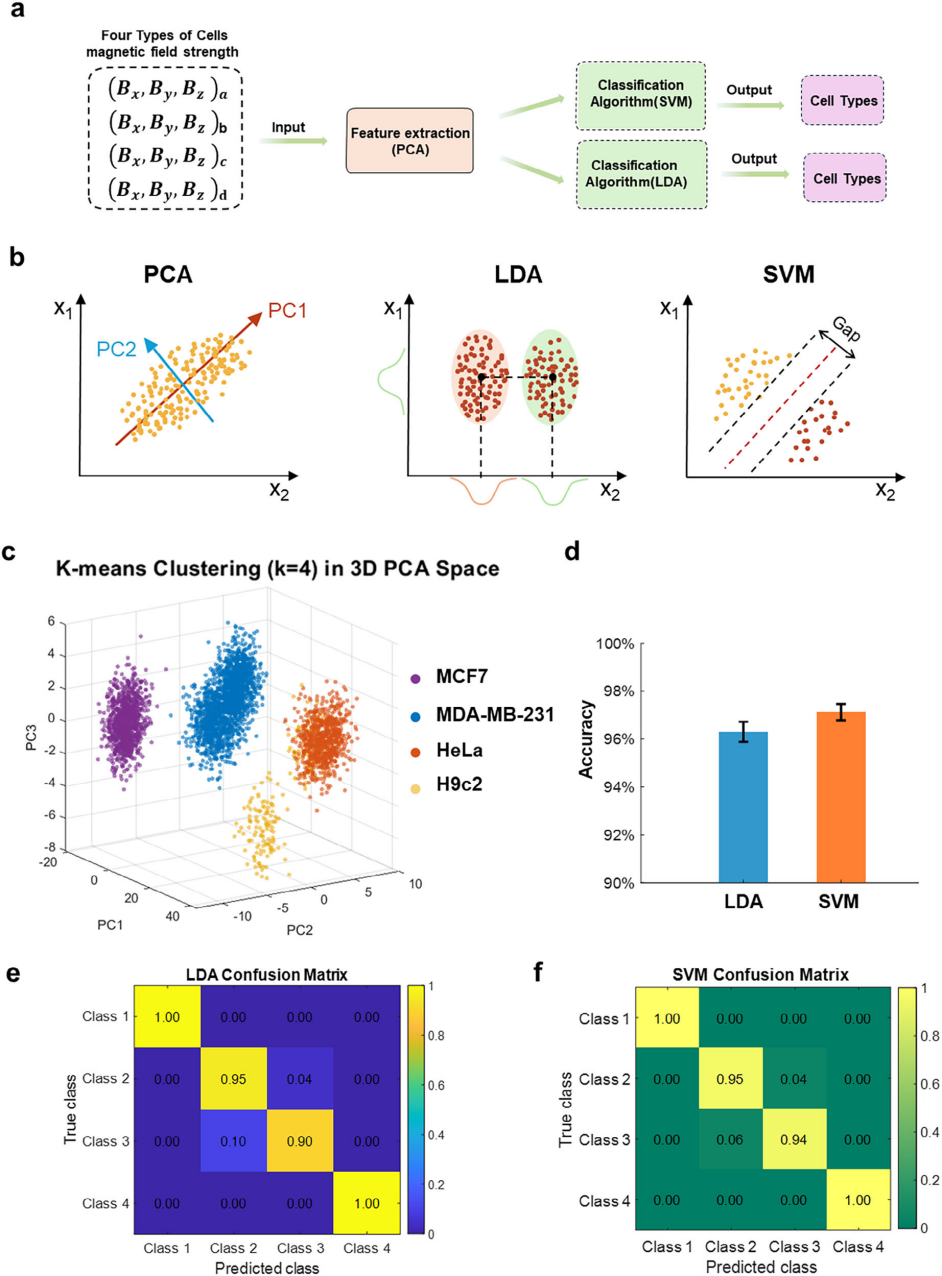
Machine learning‐based cell classification from magnetic field features. (a) Schematic of the classification pipeline: 3D magnetic field data were first processed via principal component analysis (PCA) for feature extraction, followed by classification using two machine learning algorithms, support vector machine (SVM) and linear discriminant analysis (LDA). Cell type was used as the classification output. (b) Algorithmic illustration showing the principles of PCA‐based dimensionality reduction and the core mechanisms of LDA and SVM for decision boundary construction. (c) PCA followed by K‐means clustering was applied for unsupervised grouping of four cell types based on magnetic field features. (d) Classification accuracy comparison between LDA and SVM. (e,f) Confusion matrices depicting the classification performance of LDA (e) and SVM (f), respectively. Class 1‐HeLa, Class 2‐H9c2, Class 3‐MDA‐MB‐231, Class 4‐MCF7.

To evaluate the capability of MagMI in discriminating cell types based on their biomechanical activity, we implemented a machine learning pipeline that classifies cellular phenotypes from spatiotemporal magnetic field signatures (Figure 6a). Specifically, time‐resolved magnetic field vectors(*B_x_*,*B_y_*,*B_z_*)from four distinct cell lines (MCF7, MDA‐MB‐231, HeLa, and H9c2) were processed through principal component analysis (PCA) to extract dominant variance features, which were subsequently used as inputs to two supervised classifiers: linear discriminant analysis (LDA) and support vector machine (SVM). As illustrated in Figure 6b, PCA identified principal axes (PC1, PC2) that encapsulated the predominant variance within the magnetic data. Both LDA and SVM classifiers constructed optimal decision boundaries in the reduced feature space. SVM's hyperplane yielded wider separation margins compared to LDA's linear discriminants, suggesting better generalization.

Unsupervised K‐means clustering (k = 4) applied in the PCA‐reduced space revealed four clear, compact clusters corresponding to each cell line (Figure 6c). Notably, MDA‐MB‐231 and H9c2 formed well‐separated clusters, indicating distinct biomechanical signatures arising from their differential cytoskeletal and proliferative behavior. Supervised classification results confirmed this separability: SVM achieved higher overall accuracy (∼97.2%) compared to LDA (∼95.8%), highlighting its superior boundary optimization in nonlinear subspaces (Figure 6d). Confusion matrices (Figure 6e,f) further validated the models’ robustness. SVM achieved perfect classification (>99 %) for MCF7 and H9c2, and only a minor misclassification between H9c2 and MDA‐MB‐231. LDA also performed well but showed slightly higher off‐diagonal errors in HeLa prediction, suggesting lower sensitivity to inter‐class margins.

These results demonstrate that the MagMI platform not only captures proliferation dynamics but also encodes sufficient discriminative biomechanical information for robust, label‐free classification of cell identity. The synergistic application of PCA with both unsupervised and supervised algorithms reveals that magnetic field perturbations induced by cellular activity are phenotype‐specific and can be decoded with high fidelity. This establishes MagMI as a multi‐functional diagnostic tool, enabling real‐time cell typing, phenotypic mapping, and downstream decision‐making in drug screening and regenerative engineering workflows, all without reliance on optical labeling or endpoint assays.

To translate the raw magneto‐mechanical signals into actionable biological insights and enable closed‐loop experimentation, we developed a dedicated software suite, MagVizio. This integrated platform performs real‐time data processing (including filtering and feature extraction), dynamic 3D visualization of magnetic field perturbations, and seamless interfacing with our machine learning models. MagVizio thus serves as the critical link between physical sensing and biological interpretation, allowing for immediate prediction, phenotype classification, and experimental feedback. Detailed descriptions of the software architecture, interface, and functionalities are provided in the Supplementary Note (see also Figures S9–S16)

## 3 Discussion

The MagMI platform represents a fundamental advance in real‐time, non‐invasive monitoring of cellular behaviors within 3D microenvironments by integrating magneto‐mechanical sensing with machine intelligence. Our results demonstrate that this system not only decodes proliferation dynamics and drug responses with high accuracy but also discriminates cell phenotypes based on intrinsic biomechanical signatures—all without optical labels or invasive electrodes. These capabilities directly address long‐standing limitations in current 3D cell culture monitoring, where non‐destructive, longitudinal readouts of mechanical dynamics remain scarce despite their critical role in physiological processes such as tumor invasion and drug resistance [24, 25].

Traditional endpoint assays (e.g., MTT, CCK‐8) and fluorescence‐based methods preclude continuous observation and often introduce artifacts through sample destruction or phototoxicity. Although real‐time optical techniques such as Raman spectroscopy or lens‐free computational imaging offer alternatives, they struggle with photon scattering in dense or opaque matrices, require complex computational processing, and may alter cell behavior due to prolonged light exposure [26, 27]. Electrical impedance sensing (ECIS), while label‐free, lacks spatial resolution for subcellular force mapping and is poorly suited to true 3D microenvironments due to its reliance on surface‐confined electrodes [28, 29]. In contrast, MagMI leverages magnetic field perturbations induced by nanoscale cellular forces, enabling continuous, label‐free monitoring in physiologically relevant conditions without optical interference. This offers a distinctive advantage over existing methods, particularly for long‐term studies in optically opaque systems such as hydrogels or high‐density spheroids.

A core innovation of our platform is the fusion of engineered magneto‐mechanical materials with data‐driven modeling. It is important to distinguish that our system does not directly quantify the molecular events of cell proliferation. Instead, it operates on the fundamental biomechanical principle that cell proliferation within a physically confined space, such as our micropillar array, inevitably generates nanoscale expansive and pushing forces. MagMI directly detects these proliferation‐induced mechanical forces through the resultant magnetic field perturbations. The machine learning models are then trained to establish a robust correlation between the spatiotemporal patterns of these mechanical signals and independently validated measures of cell population growth (e.g., OD values from CCK‐8 assays). Therefore, the platform intelligently infers proliferation dynamics by decoding the biomechanical signatures that are a direct physical consequence of proliferation. The soft magnetic micropillars transduce nanoscale cellular forces into spatially encoded magnetic signals, while machine learning decodes these high‐dimensional spatiotemporal patterns without relying on explicit physical models or handcrafted features. This approach aligns with emerging trends in AI‐enhanced biological sensing [30] but goes further by closing the loop between physical sensing and biological interpretation via the integrated MagVizio software platform. Where recent studies have emphasized the need for non‐destructive monitoring in 3D cultures [31], MagMI delivers a comprehensive solution that spans from real‐time sensing to predictive modeling and user‐friendly data visualization.

Notably, MagMI also distinguished cell phenotypes through magnetic‐field‐derived biomechanical signatures. Principal component analysis revealed separable feature spaces, and both unsupervised clustering and supervised classification (SVM >97% accuracy) confirmed that spatiotemporal magnetic profiles are phenotype‐specific and discriminative. This highlights the platform's capacity for real‐time cell typing and label‐free phenotypic mapping. These findings corroborate growing evidence that mechanical cues are highly discriminative of cell state and function [32, 33, 34]. For instance, recent research highlights that biomechanical properties influence drug sensitivity and metastatic potential in breast and prostate cancer models [35]. MagMI extends these insights by providing a quantitative, dynamic view of mechanical changes during drug exposure or phenotypic evolution, enabling earlier detection of therapeutic effects than traditional metabolic or optical assays.

Moreover, the platform's performance in predicting proliferation curves and drug responses across multiple cell lines highlights its robustness and generalizability. Under doxorubicin treatment, magnetic field decay patterns reflected dose‐dependent cytotoxicity and were reliably decoded into suppressed growth curves. The models maintained predictive performance even under increased biological heterogeneity, suggesting applicability for early‐stage, non‐invasive drug response assessment. Importantly, low RMSLE (0.041–0.042) values indicate sensitivity to subtle changes, supporting the platform's use in fine‐resolution pharmacodynamics. This capability is particularly relevant for preclinical drug screening, where conventional methods often fail to predict clinical outcomes due to oversimplified culture conditions [36, 37]. By capturing mechanical heterogeneity in 3D microenvironments, MagMI offers a more pathologically relevant model for evaluating drug efficacy and mechanisms of action. To enhance accessibility and interpretability, the MagVizio software integrates signal filtering, 3D field visualization, and model inference into a modular graphical user interface (GUI), supporting interactive exploration and low‐barrier deployment. Together, these results establish MagMI as a scalable, label‐free diagnostic framework for real‐time analysis of proliferation, drug response, and phenotype classification in 3D biological systems.

Looking forward, MagMI's closed‐loop architecture—from sensing to analysis via MagVizio—sets a new standard for intelligent experimentation in biomedical research, echoing the broader movement toward AI‐driven automation in scientific discovery. Scaling MagMI to high‐density arrays is a key direction for high‐throughput applications, requiring solutions to two core challenges: magnetic crosstalk and signal noise. For 96‐well plate compatibility (e.g., 24×16 Hall sensor arrays), the miniaturization and sensor layout of Hall sensor arrays can reduce the magnetic overlap between adjacent sensors, which requires advanced printed circuit board (PCB) design and manufacturing. Circuit miniaturization with low‐noise amplifiers and differential signal acquisition mitigates noise from large‐scale data integration. The modular hardware design (separate sensor, signal conditioning, and data transmission layers) and reusable machine learning models facilitate seamless scaling. High‐density arrays would enable simultaneous monitoring of mechanical heterogeneity across cell lines or 3D microenvironments, accelerating drug screening by quantifying “dose‐mechanical response” curves for dozens of conditions in parallel—representing a ≈20‐fold efficiency improvement over current 4×3 arrays. The platform's compatibility with standard multi‐well plates and scalable sensing design facilitates integration into high‐throughput screening pipelines.

Future applications could couple with other sensing modalities, such as electrophysiological recording. While MagMI provides unique access to nanoscale biomechanical forces, coupling it with microelectrode arrays (MEAs) for extracellular and intracellular recording would enable truly multimodal correlative analysis. This synergy would be particularly transformative for studying electrogenic cells, such as cardiomyocytes and neurons. For example, combining MagMI with high‐density microelectrode arrays (MEAs) [38, 39] could simultaneously capture mechanical forces (via magnetic signals) and electrical activities (e.g., action potentials, field potentials), enabling correlative studies of mechanotransduction and ionic signaling. In drug screening on cardiomyocytes, one could simultaneously record the electrophysiological profile (e.g., action potential shape and firing rate) and the concomitant mechanical contractile forces, providing a holistic view of drug‐induced cardiotoxicity. Such multimodal integration could provide deeper insights into drug responses—for instance, distinguishing drugs that specifically inhibit proliferation‐related forces from those that alter both mechanical and electrical functions. In vivo, miniaturized MagMI arrays might be combined with flexible neural probes [40, 41] to monitor neurite growth mechanics alongside neural circuit activity, advancing studies of neural regeneration. It could unveil new insights into neuro‐electro‐mechanical coupling. The primary engineering challenge will be the co‐design and co‐fabrication of magneto‐mechanical pillars and transparent microelectrodes on a shared substrate to minimize crosstalk and ensure biocompatibility. Successfully navigating this integration would establish a powerful platform for deciphering the intricate interplay between electrical and mechanical signaling in physiological and pathological states.

Nevertheless, challenges remain. Long‐term biocompatibility of magnetic materials, computational demands for real‐time processing of large datasets, and standardization of model validation across laboratories will require further attention. Additionally, combining magnetic mechanical data with other modalities—such as biochemical or genomic readouts—could unlock multi‐scale insights into cell behavior.

## 4 Conclusions

Overall, this study establishes MagMI as a robust and scalable platform that leverages machine learning to decode complex cellular behaviors from magnetic field perturbations in a fully label‐free, non‐invasive manner. Machine learning models demonstrated strong predictive performance across diverse cell types and drug conditions, extracting biologically relevant features from high‐dimensional spatiotemporal data with minimal overfitting. Both regression and classification tasks confirmed that magneto‐mechanical signatures encode rich, phenotype‐specific information, enabling accurate prediction of proliferation dynamics and real‐time discrimination of cell identity.

MagMI bridges a critical gap in 3D cellular analytics by providing a label‐free, mechanically sensitive, and AI‐powered platform for dynamic cellular monitoring. Its ability to decode proliferation, drug response, and phenotypic identity through biomechanical signatures positions it as a transformative tool for accelerating drug discovery, personalized medicine, and fundamental research in cancer biology and beyond. It is expected to accelerate the emergence of autonomous and intelligently automated experimental platforms.

## 5 Methods and Materials

### 5.1 Materials

The neodymium‐iron‐boron (NdFeB) particles were purchased from Magnequench, China. Polydimethylsiloxane (PDMS) base and curing agent (Sylgard 184 kit) were purchased from Dow Corning, USA.

### 5.2 Fabrication of Magnetic Micropillar Arrays

The silicon master mold for casting the micropillar arrays was fabricated using standard photolithography and deep reactive ion etching (DRIE) processes. Specifically, a 4‐inch silicon wafer was first cleaned with acetone, isopropanol, and deionized water, followed by oxygen plasma treatment to ensure surface cleanliness and hydrophilicity. A positive photoresist was then spin‐coated onto the wafer, resulting in a thickness of approximately 8 µm. The coated wafer was soft‐baked at 100°C for 1 min. Subsequently, the wafer was exposed to UV light through a photomask defining the 10 µm diameter circular patterns arranged in the desired array layout, using a mask aligner. Post‐exposure baking was conducted at 110°C for 1 min. The pattern was then developed in developer for 60 s, revealing the silicon substrate in the exposed areas. The patterned wafer underwent a descumming process via oxygen plasma to remove any residual photoresist. The silicon was then etched using the DRIE process to create cylindrical holes with a target depth of 30 µm, which defines the height of the subsequent pillars. The remaining photoresist was thoroughly stripped using acetone and oxygen plasma, resulting in a clean silicon master mold with negative features of the micropillar array. Finally, the silicon mold was silanized with (tridecafluoro‐1,1,2,2‐tetrahydrooctyl) trichlorosilane vapor for 4 h under vacuum to facilitate the demolding of the polydimethylsiloxane (PDMS) replica. PDMS, curing agent, and NdFeB magnetic powder (particle size < 5 µm) were mixed at a mass ratio of 10:1:10 (PDMS base: curing agent: NdFeB). The mixture was degassed under vacuum for 5 min and cast onto the silicon template. The entire assembly was cured at 80°C for 2 h. After curing and cooling, the composite PDMS/ NdFeB substrate containing the micropillar array was demolded by peeling it from the template. The substrate was magnetized using a commercial magnetizer (MA‐3030, Jiu Juok, Shenzhen, China) with a constant magnetic field of 3 T. The resulting magnetic micropillars exhibited a diameter of 10 µm and a height of 30 µm. (The detailed production process can be seen in Figure S8.) After that, circle patterns were engraved on the film using a laser engraving machine (LPKF ProtoLaser U4, LPKF Laser& Electronics AG, Germany).

### 5.3 Cell Culture and Cell Proliferation Analysis

MDA‐MB‐231 (RRID: CVCL_0062), HeLa (RRID: CVCL_0030), MCF7 (RRID: CVCL_0031), and H9c2 rat cardiomyoblasts (RRID: CVCL_0286) were procured from American Type Culture Collection (ATCC). Cell lines were tested and authenticated by morphology and growth rate, and were confirmed to be mycoplasma‐free. Cells were cultured on UV‐sterilized magnetic micropillar substrates in Dulbecco's Modified Eagle Medium (DMEM; Gibco) supplemented with 10% fetal bovine serum (FBS; Gibco), 100 U/mL penicillin, and 100 µg/mL streptomycin (Gibco) at 37°C in a 5% CO_2_ humidified atmosphere. Prior to cell seeding, substrates underwent UV sterilization (254 nm, 60 min) followed by incubation with 5 µg/mL fibronectin (Solarbio, Cat #F8180; diluted in PBS) for 1 h at 37°C.

Cell viability was assessed after 48 h of culture using a LIVE/DEAD Viability/Cytotoxicity Kit (Invitrogen, Cat #R37601). Following manufacturer protocols, cells were incubated with calcein‐AM and ethidium homodimer‐1 in phosphate‐buffered saline (PBS) for 30 min at 37°C, then imaged using an inverted fluorescence microscope. Cell proliferation was quantified via CCK‐8 assay (Solarbio, Cat #CA1210).

### 5.4 Cell Staining and Morphological Observations

A warm PBS solution was used to wash cells. Then the cells were fixed in 4% paraformaldehyde for 30 min and permeabilized with 0.5% v/v Triton X‐100 with 1% bovine serum albumin (BSA) for another 30 min. In order to label the filamentous actin (F‐actin), we incubated the cells with 1 µg/mL Phalloidin‐FITC (CA1620, Solarbio, Beijing, China) at room temperature for 60 min. We labeled cell nuclei with 4′,6‐diamidino‐2‐phenylindole (DAPI) (S2110, Solarbio, Beijing, China) for 5 min. All samples were imaged using a Zeiss LSM 710 confocal microscope.

### 5.5 Characterizations

The SEM and EDS images were obtained by field‐emission scanning electron microscopy (FE‐SEM, Carl Zeiss, Germany). The magnetic hysteresis loops (M‐H curve) were measured by a physical property measurement system (PPMS). The elastic modulus of PDMS/NdFeB was determined by the commercial motorized platform (ESM303, Mark‐10 Corporation, USA)

### 5.6 Simulation of Magnetic Field

The software, COMSOL Multiphysics 5.6, was employed to simulate the magnetic field around the magnetic micropillar array. N54 (sintered NdFeB) was given to the model. The air atmosphere was set with a dimension of 400 µm × 400 µm. The mesh was controlled by the physics interfaces with a regular size. Magnetic field (no current) module was employed to trace the magnetic scalar potential and magnetic flux density, with the governing equations ***H*** = −∇ ***V***_m_ and ∇∙ ***B*** = 0, where ***H*** is the magnetic field vector, ***B*** = µ_0_µ_r_ ***H*** is the magnetic flux density vector, and ***V***_m_ the magnetic scalar potential. The boundary conditions were set as ***n∙B*** = 0. The initial value of the magnetic scalar potential was set as 0. The constitutive relation between the magnetic field and magnetization was governed by ***B*** = µ_0_(***H*** + ***M***), where ***M*** is the magnetization vector.

### 5.7 Machine Learning Regression Pipeline: PCA‐PLSR With K‐Fold Cross‐Validation

To quantitatively decode cellular responses from high‐dimensional magnetic field data, we implemented a machine learning pipeline combining principal component analysis (PCA) [42] with partial least squares regression (PLSR) [43], evaluated under K‐fold cross‐validation. This approach enables robust performance assessment while mitigating overfitting, particularly in data‐limited or high‐noise biological scenarios.

Input data consisted of a feature matrix $X\in R^{N\times d}$, representing the spatiotemporal magnetic field descriptors, and a corresponding target matrix $Y\in R^{N\times m}$ typically containing the cellular absorbance (OD value) measured in parallel by the CCK‐8 assay, which serves as a standard metric for cell viability and proliferation. To generate the proliferation curves, the OD values for each experiment were normalized to their initial value at Time = 0, defining the “Relative Proliferation” on the Y‐axis of the prediction plots (e.g., Figures 3 and 4). The machine learning model was thus trained to learn the functional mapping from the spatiotemporal magnetic field descriptors (X) to these normalized proliferation metrics (Y). The predicted proliferation curves are the model's outputs when fed the time‐series magnetic field data. If the data were row‐oriented, both 𝑋 and 𝑌 were transposed to ensure consistent formatting. The number of rows in 𝑋 and 𝑌was asserted to match, reflecting paired observations. The feature matrix 𝑋 was standardized using z‐score normalization to a zero mean and unit variance. Dimensionality reduction was then performed via PCA, retaining the top 𝑝 principal components based on explained variance.

A 𝐾‐fold cross‐validation scheme was employed to partition the dataset into training and testing subsets. For each fold 𝑘 = 1,…,𝐾, the PLSR model was trained on the training partition using the reduced features *X^train^* and corresponding standardized targets *Y^train^*, with the number of latent variables fixed to 𝑝, matching the number of principal components. Model coefficients $\beta \in R^{(p+1)\times m}$ were estimated using the plsregress function. Predictions were then generated for the test partition using:

$$\hat{Y_{z}}=\left[1,X^{test}\right]\times \beta$$

and rescaled to the original target space via inverse standardization using the training mean and standard deviation. The predicted values $\hat{Y_{z}}$ were compared against the true values *Y^test^* to compute performance metrics.

### 5.8 Supervised Classification With K‐Fold Cross‐Validation

To evaluate the discriminative capacity of magnetic‐field‐derived features in identifying cell phenotypes, we implemented a supervised classification pipeline using either multiclass support vector machines (SVM) [44] or linear discriminant analysis (LDA) [45]. Classification performance was quantified using fivefold cross‐validation, ensuring generalization and robustness across phenotypic variations.

The full dataset was composed of class‐wise feature matrices *A, B, C, and D*, each corresponding to a distinct cell phenotype. These were concatenated into a single feature matrix $X\in R^{N\times d}$, with a corresponding label vector *Y* ∈ {1, 2, 3, 4}^*N*^, where *N* is the total number of samples and *d* is the number of extracted features.

We adopted a fivefold stratified cross‐validation scheme to partition the dataset into training and testing subsets. At each fold *i* ∈ {1, 2, 3, 4, 5}, the model was trained on 80% of the data and tested on the remaining 20%.

Two classifiers were independently evaluated: Multiclass Support Vector Machine (SVM): Implemented using the one‐vs‐one error‐correcting output code (ECOC) strategy via fitcecoc. LDA: Implemented using fitcdiscr, which constructs linear boundaries between classes based on class‐specific covariances.

At each fold, the selected model was trained on *X^train^* and *Y^train^*, and then used to predict labels for *X^test^* Predicted labels *Y^test^* were compared against ground truth to calculate fold‐specific accuracy:

$$\mathrm{Accurac}y_{i}=\frac{1}{n_{i}}\sum_{j=1}^{n_{i}}1\left(\hat{y_{j}}=y_{j}\right)$$

where *n_i_* is the number of test samples in fold *i*, and 1(⋅) is the indicator function.

The final performance was reported as the mean classification accuracy across all fivefolds:

$$\mathrm{Mean}\;\mathrm{Accuracy}=\frac{1}{5}\sum_{i=1}^{5}\mathrm{Accurac}y_{i}$$

All computations were performed in MATLAB R2023a using built‐in machine learning functions. Classifier performance was additionally visualized via confusion matrices to reveal misclassification patterns and inter‐class separability.

### 5.9 Statistical Analysis

The data were expressed as the “mean ± standard deviation”. Error bars in all figures are the standard deviations obtained from at least five independent measurements unless otherwise stated. All the data were analyzed and performed by Origin Software and MATLAB.

## Author Contributions

Y.Q. and Y.W. performed in conceptualization, formal analysis, methodology, and writing – original draft. B.Z. performed in conceptualization, methodology, investigation, and visualization. S.D. performed in supervision, methodology, and visualization. Y.Z. performed in funding acquisition, supervision, writing – review, and editing.

## Conflicts of Interest

The authors declare no conflict of interest.

## Supporting information




**Supporting File**: advs73458‐sup‐0001‐SuppMat.docx.

## Data Availability

The data that support the findings of this study are available from the corresponding author upon reasonable request.
