# Supplementary material for: An Intelligent Magneto‐Mechanical Platform for Cellular Sensing in 3D Microenvironments
Source: Adv Sci (Weinh). 2025 Dec 22;13(17):e19132. doi: 10.1002/advs.202519132 (PMC13042406; doi:10.1002/advs.202519132)
Supplement: Supplementary file 1 — Supporting File: advs73458‐sup‐0001‐SuppMat.docx. [file ADVS-13-e19132-s001.docx]

*Supplementary Information*

*For*

**An Intelligent Magneto-Mechanical Platform for Cellular Sensing in 3D Microenvironments**

Yue Quan^#^, Yuxin Wang^#^, Sen Ding^*^, Bingpu Zhou, Yinning Zhou^*^

Joint Key Laboratory of the Ministry of Education, Institute of Applied Physics and Materials Engineering, University of Macau, Avenida da Universidade, Taipa, Macau

^#^These authors contributed equally to this work.

* Corresponding author email:

Yinning Zhou: [ynzhou@um.edu.mo](mailto:ynzhou@um.edu.mo);

Contents

[Supplementary Algorithm Data 39](#_Toc216014773)

[Supplementary Figures 44](#_Toc216014774)

[Supplementary Note 52](#_Toc216014775)

# *Supplementary Algorithm Data*

**Algorithm 1 Magnetic Processing Pipeline**

**Input:** data = {Cell_mag}, nPts = 10.^6, nFiles = 5, nRep = 12

**Output:** Bmag (nTot×nPts),

per-axis stacks (X_mag_normalize,Y_mag_normalize,Z_mag_ normalize)

**1. Init**

nTot ← nFiles * nRep

X_mag, Y_mag, Z_mag ← zeros(nTot, nPts)

**2. Load & split channels**

**for** k in 1..nFiles **do**

X_mag ← data(:, 1:3:end); Y_mag ← data(:, 2:3:end); Z_mag ← data(:, 3:3:end)

**end**

**3. Vector magnitude**

Bmag ← sqrt( X_mag.^2 + Y_mag.^2 + Z_mag.^2 )

**4. Mean ± SD band for a chosen tensor B (B = Bmag)**

μ ← mean(B, dim=1)

σ ← std(B, 0, dim=1)

upper ← μ + σ; lower ← μ − σ

tHours ← linspace(0, 48, length(μ))

**5. Single-trial extraction & preprocessing (X axis)**

Xraw ← double(X_mag)

Xout ← filloutliers(Xraw, method='linear', window='movmedian', w=101)

X_mag_normalize ← normalize(Xout, "range")

**6. Single-trial extraction & preprocessing (Y axis)**

Yraw ← double(Y_mag)

Yout ← filloutliers(Yraw, method='linear', window='movmedian', w=101)

Y_mag_normalize ← normalize(Yout, "range")

**7. Single-trial extraction & preprocessing (Z axis)**

Zraw ← double(Z_mag)

Zout ← filloutliers(Zraw, method='linear', window='movmedian', w=101)

Z_mag_normalize ← normalize(Zout, "range")

**return** Bmag, X_mag_normalize, Y_mag_normalize, Z_mag_normalize, μ, σ, upper, lower, t

**Algorithm 2 PCA-PLSR with K-Fold Cross Validation**

**Input:**

X ← feature matrix

Y ← target matrix

p ← number of principal components

K ← number of folds for cross-validation

**Output:**

metrics for each fold: [RMSE, MAE, RMSLE, R², R²adj]

**Procedure:**

**1. Preprocess input:**

**if** X or Y are row-oriented → transpose

**assert** number of rows(X) = number of rows(Y)

**2. Standardize features:**

Xz ← zscore(X)

**3. PCA dimensionality reduction:**

[coeff, score, explained] ← PCA(Xz, retain p comps)

Xred ← score # reduced feature space

**4. Standardize target:**

Yz ← zscore(Y)

**5. Initialize K-fold partition:**

cv ← cvpartition(N, K)

metrics ← zeros(K, 5)

**6. For each fold = 1..K:**

trIdx ← training indices

teIdx ← testing indices

# Train PLSR with p components

beta ← plsregress(Xred(trIdx,:), Yz(trIdx,:), p)

# Predict on test set

Yhat_z ← [1, Xred(teIdx,:)] * beta

Yhat ← unnormalize(Yhat_z, muY, sigmaY)

Ytrue ← Y(teIdx,:)

# Compute errors

err ← Ytrue - Yhat

mse ← mean(err²)

mae ← mean(|err|)

# RMSLE

log_true = log1p(Y_true);

log_pred = log1p(Y_hat);

rmsle = sqrt(mean((log_pred - log_true).^2));

# Coefficients of determination

SSres ← sum(err²)

SStot ← sum( (Ytrue - mean(Ytrue))² )

R² ← 1 - SSres/SStot

R²adj ← 1 - (1-R²) * (nTest-1)/(nTest-p-1)

# Store metrics

metrics(fold,:) ← [sqrt(mse), mae, rmsle, R², R²adj]

**7. Return metrics**

**Algorithm 3 Multiclass SVM/LDA with 5-Fold Cross-Validation**

**Input:**

A, B, C, D # class-wise feature matrices

**Output:**

mean 5-fold accuracy

**1. Create 5-fold partition:**

cv ← KFoldPartition(Y, K=5)

**2. Initialize:**

acc ← zero_vector(K)

**3. For each fold i in {1..K}:**

trainIdx, testIdx ← cv[i]

X_train, Y_train ← X[trainIdx,:], Y[trainIdx]

X_test, Y_test ← X[testIdx,:], Y[testIdx]

Train multiclass SVM or LDA (one-vs-one ECOC):

model ← fitcecoc(X_train, Y_train)

(model ←fitcdiscr(X_train, Y_train))

Predict:

Y_pred ← predict(model, X_test)

Measure accuracy:

acc[i] ← mean(Y_pred == Y_test)

print("Fold i Accuracy: ", 100*acc[i], "%")

**4. Report:**

print("5-fold mean accuracy: ", 100*mean(acc), "%")

**Algorithm 4 PCA + K-means with Centroid Visualization**

**Input:**

X # samples × features

k = 4 # number of clusters (can be any k)

**Output:**

idx # cluster labels for each sample

C # k × 3 centroids in PCA space

X_pca3 # samples × 3 PCA embedding

**Procedure:**

**1. Reduce to 3D via PCA:**

scores, coeff ← PCA(X)

X_pca3 ← scores[:, 1..3]

**2. Cluster in 3D PCA space:**

set random seed for reproducibility

idx, C ← KMEANS(X_pca3, k) # C is k×3

**3. Visualize:**

create 3D scatter of X_pca3 colored by idx (semi-transparent markers)

label axes as PC1, PC2, PC3; set title and grid; adjust 3D view angle

**4. Return:**

idx, C, X_pca3

# *Supplementary Figures*

**
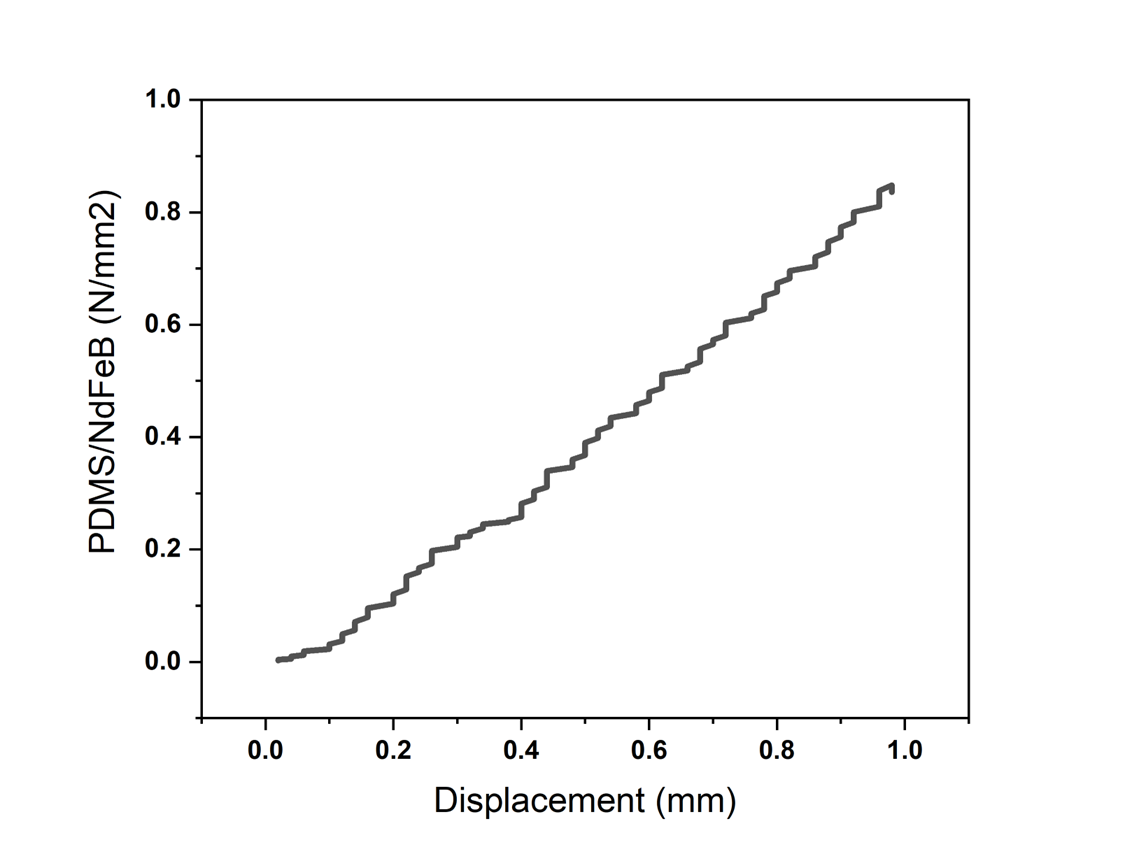
**

**Figure S1**. **The elastic modulus test of PDMS/NdFeB composite**. The formula of elastic modulus is E=FL/A∆L, F-pressure, L-model height, A-model Area, ∆L-displacement. We made the material into a 1*1*0.5 cm^3^ volume cube, applied pressure, and measured the change of displacement as the pressure increased. The slope of the curve is Young's modulus when the Y axis is F/A and the X axis is ∆L/L.


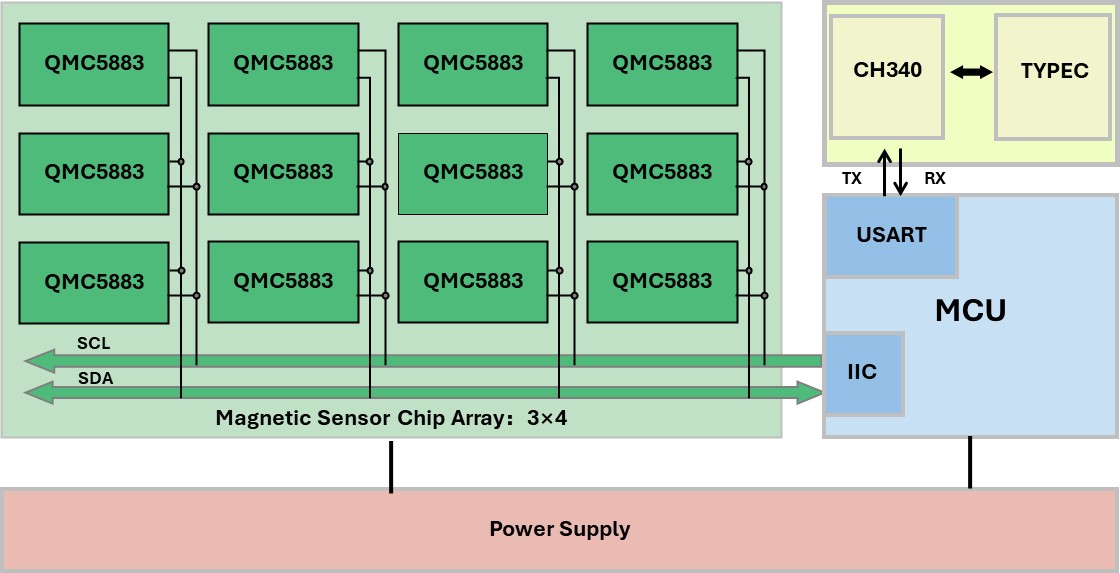


**Figure S2.** **Schematic illustration of the data collection process.**


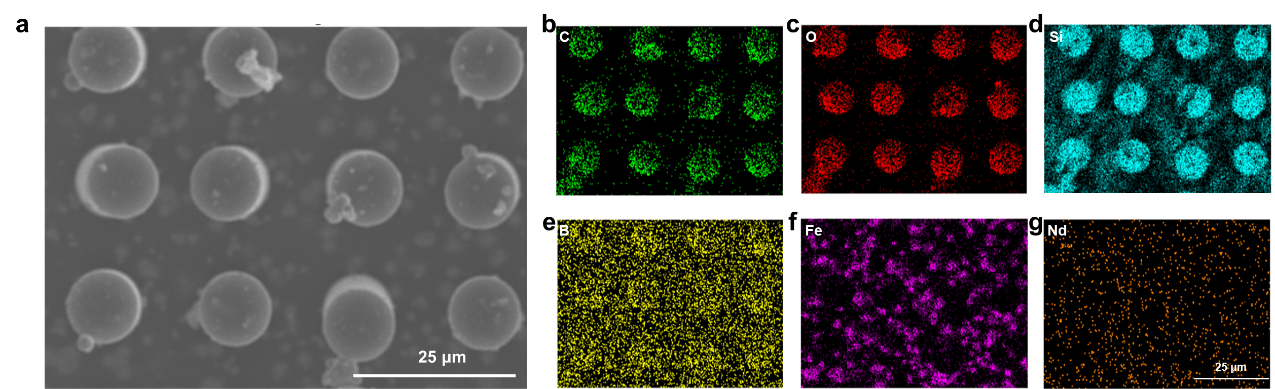


**Figure S3. Scanning electron microscopy (SEM) images and energy dispersive X-ray spectroscopy (EDS) mapping of PDMS/NdFeB micropillar array.** a, SEM image of the PDMS/NdFeB micropillar array. b-g, EDS mapping of chemical elements distributions.


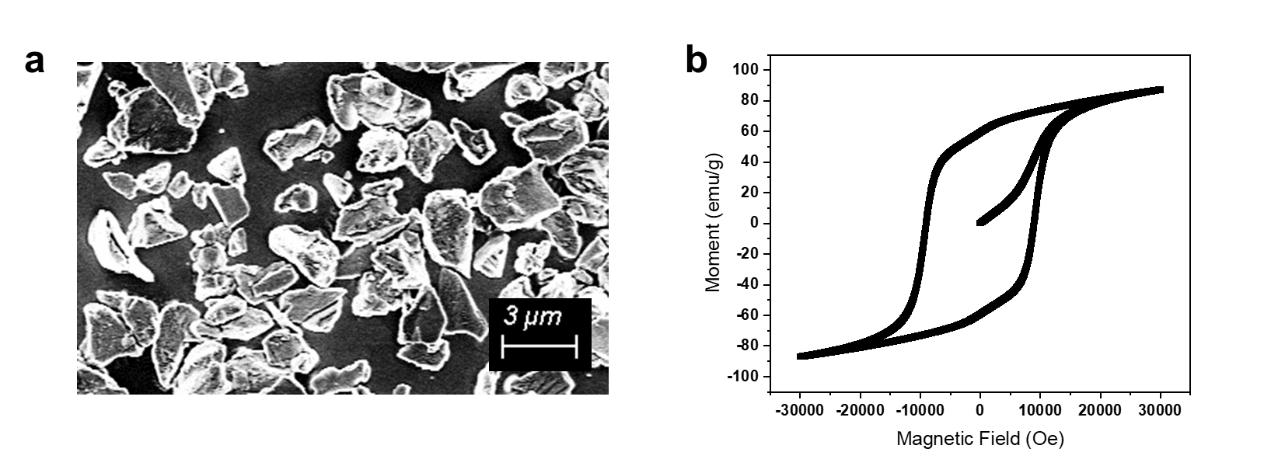


**Figure S4. Characterization of the NdFeB particles.** a, SEM image of the NdFeB particles. b, Hysteresis loop of PDMS/NdFeB micropillars with PDMS substrate.


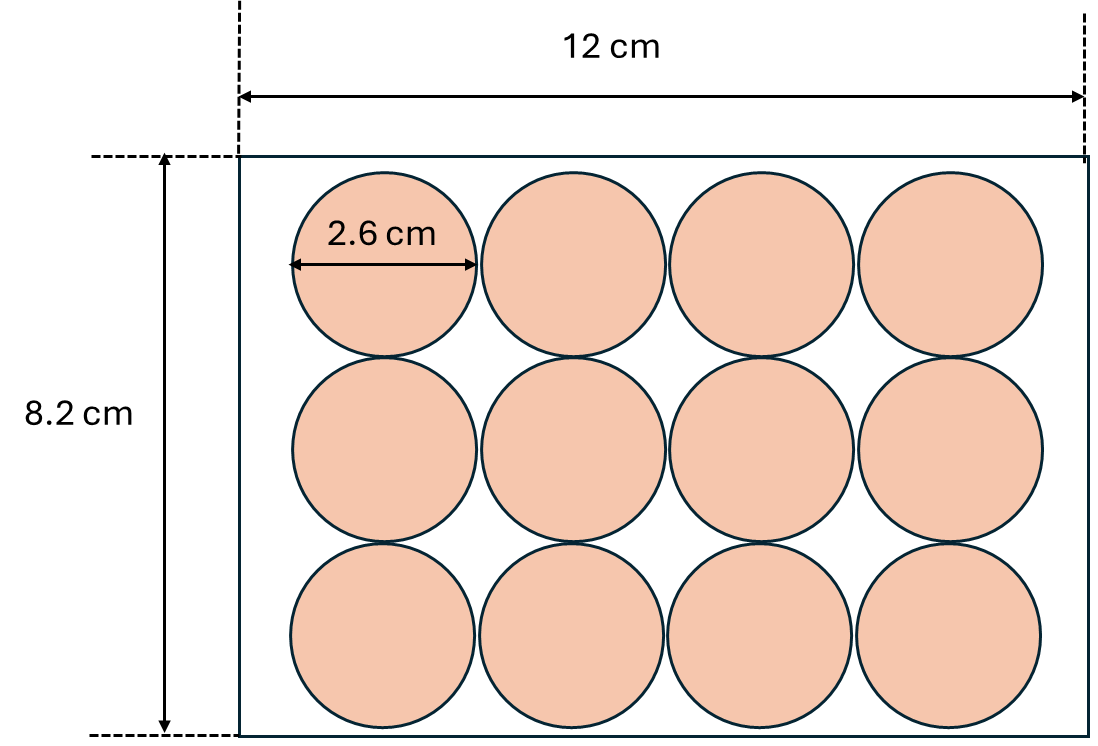


**Figure S5. The size of the Hall sensor array.**

**
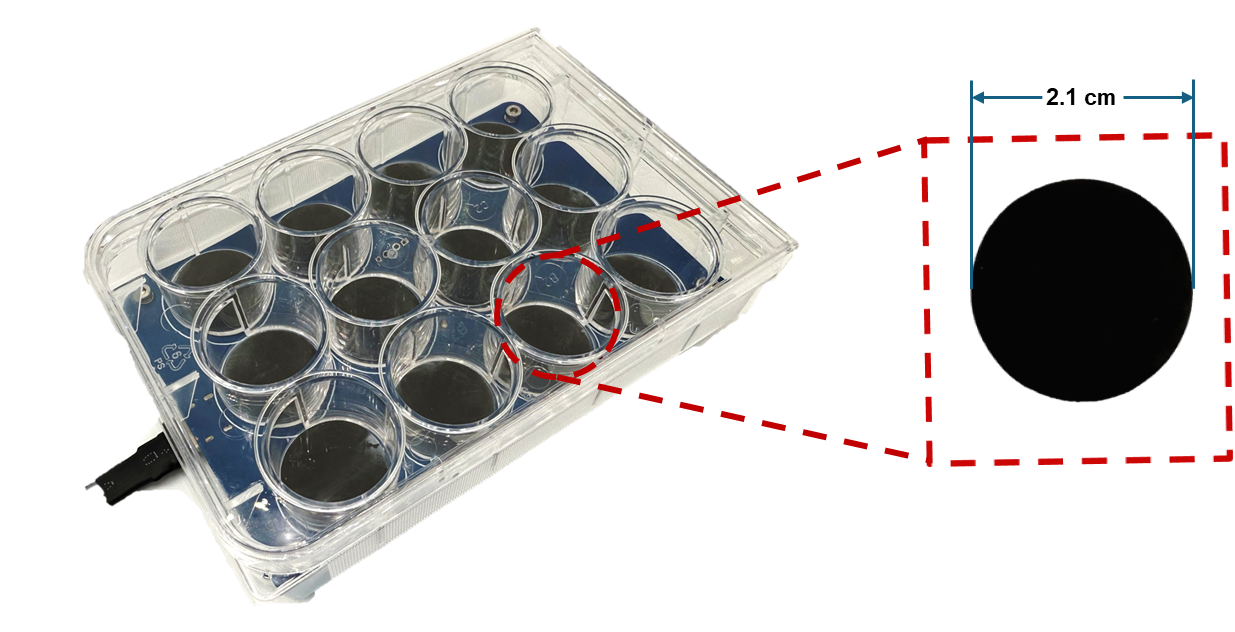
**

**Figure S6. Photograph of overall experimental device.**

**
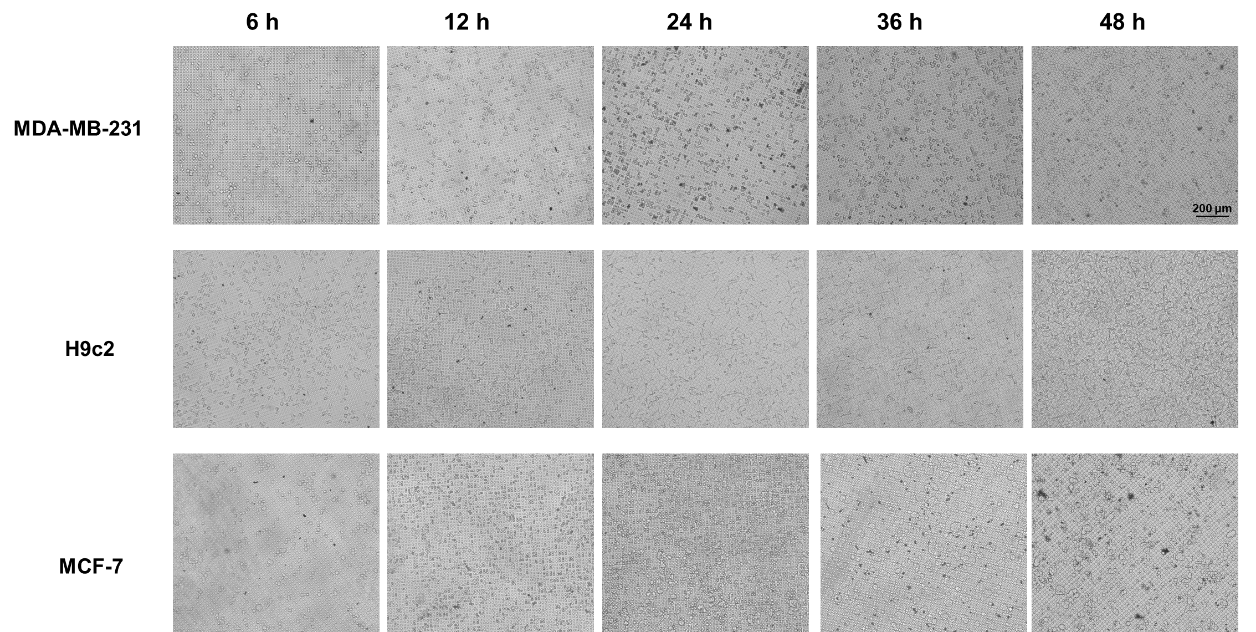
**

**Figure S7. Bright-field images of three cell types of morphology and number changes along with the time on micropillar array.**

**
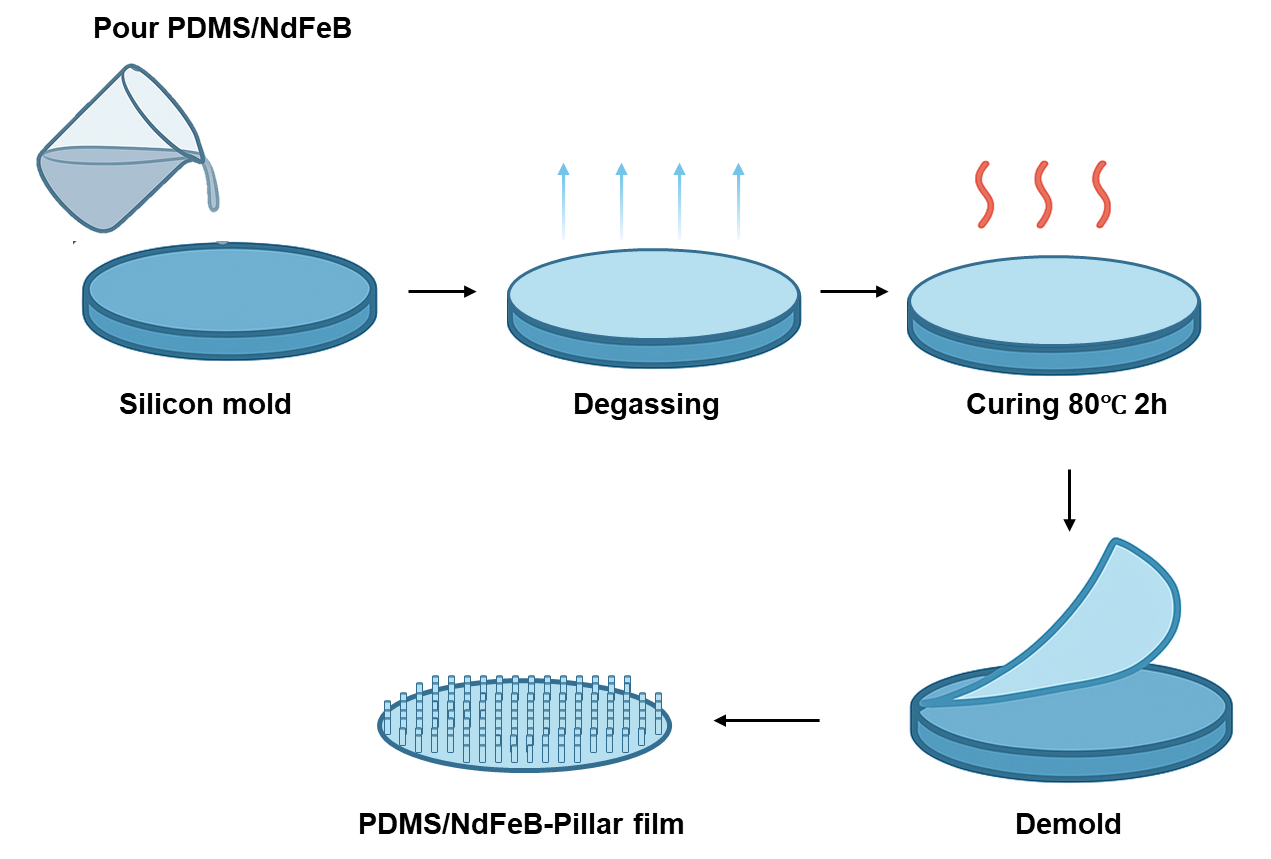
**

**Figure S8. Schematic representation of magnetic micropillar-fabrication process.**

*Supplementary Note*

**Programmable magnetic field analysis via the MagVizio software interface**

**
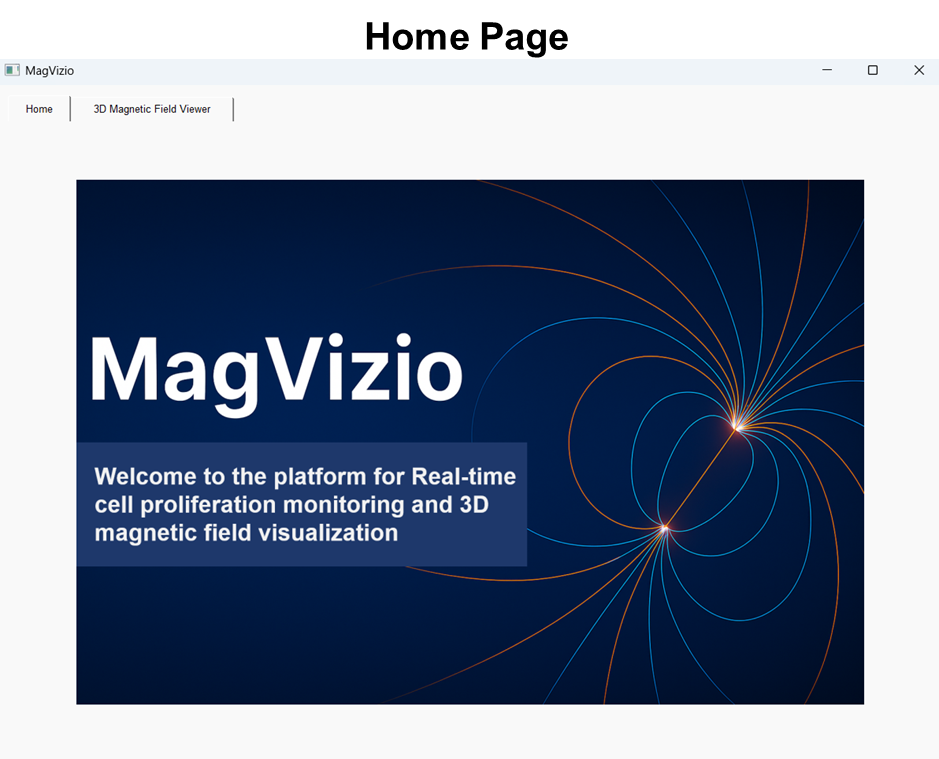
**

**Figure S9. MagVizio: an object-oriented programmable interface for magnetic field-based cellular analysis. Home interface of the MagVizio software.**

**
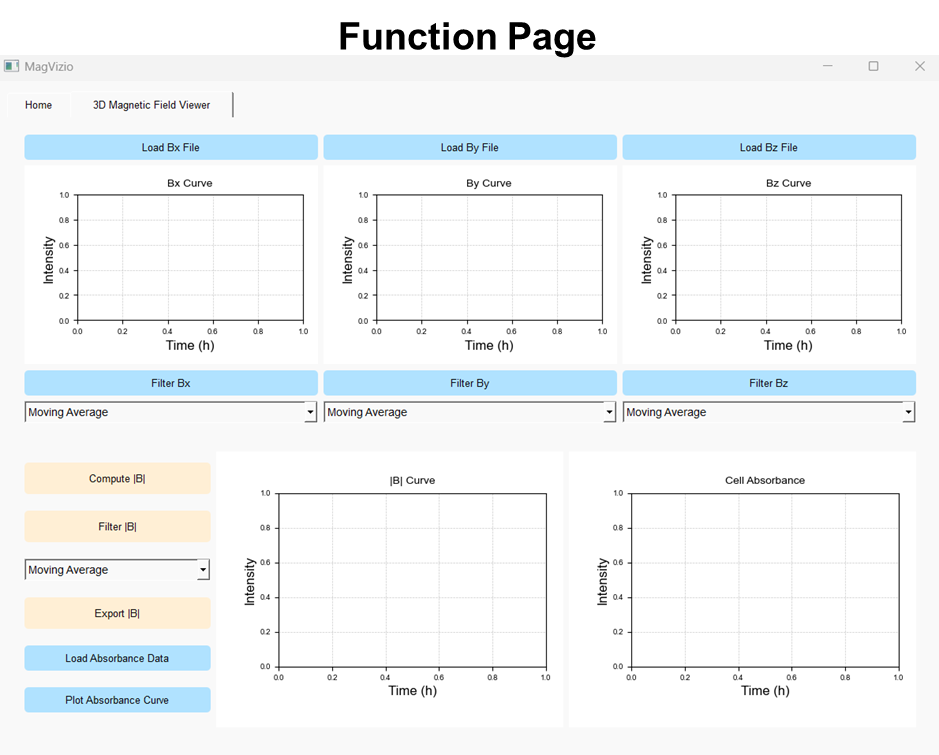
**

**Figure S10. Functional demonstration page showcasing integrated modules.**

**
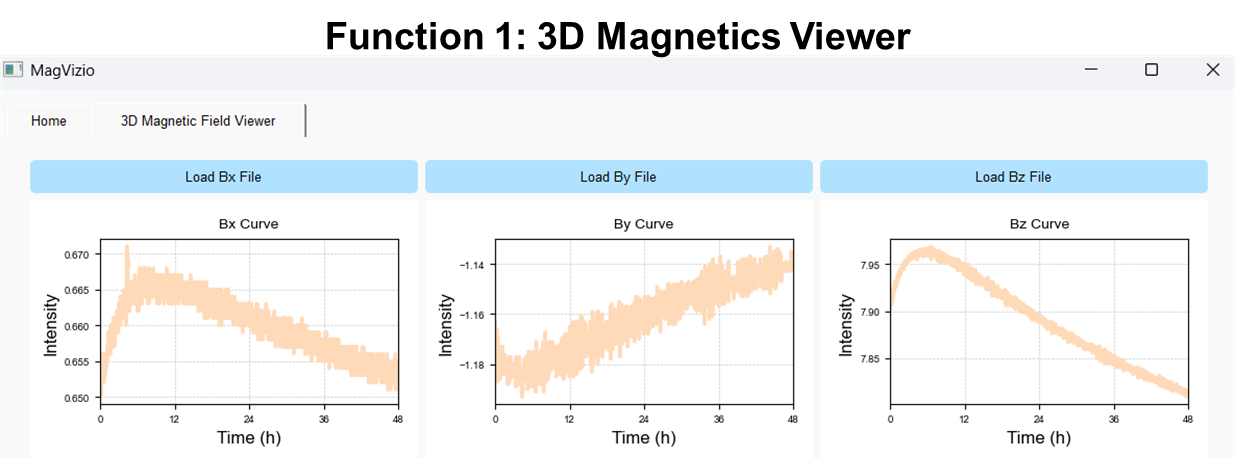
**

**Figure S11.** **3D magnetic field visualization interface enabling dynamic rendering of（**$\boldsymbol{B}_{\boldsymbol{x}}\boldsymbol{,}\boldsymbol{B}_{\boldsymbol{y}}\boldsymbol{,}\boldsymbol{B}_{\boldsymbol{z}}$**）components.**

**
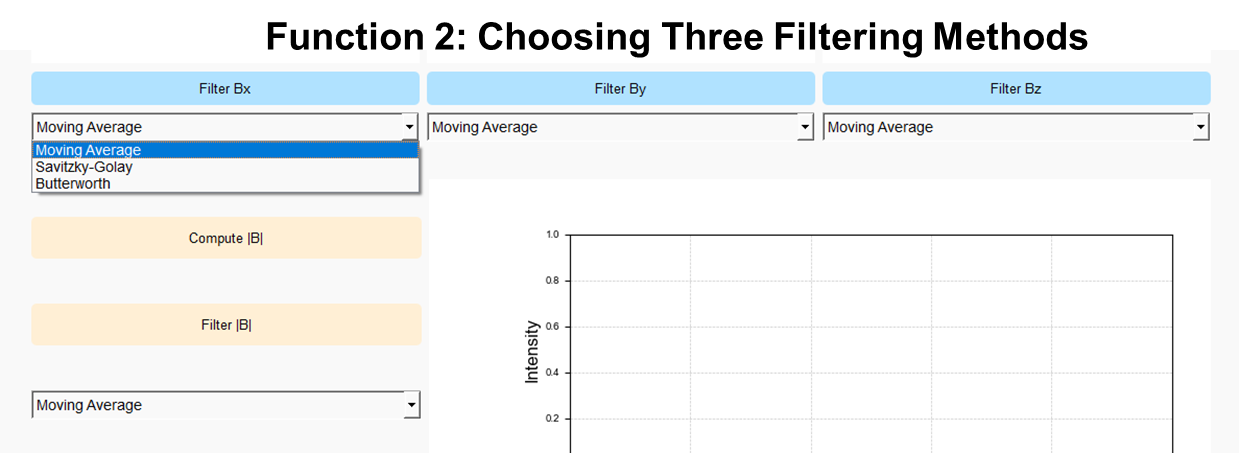
**

**Figure S12.** **Interactive panel for selecting among three built-in filtering methods: Savitzky-Golay (SG) filter, moving average smoothing, and low-pass Butterworth filtering.**

**
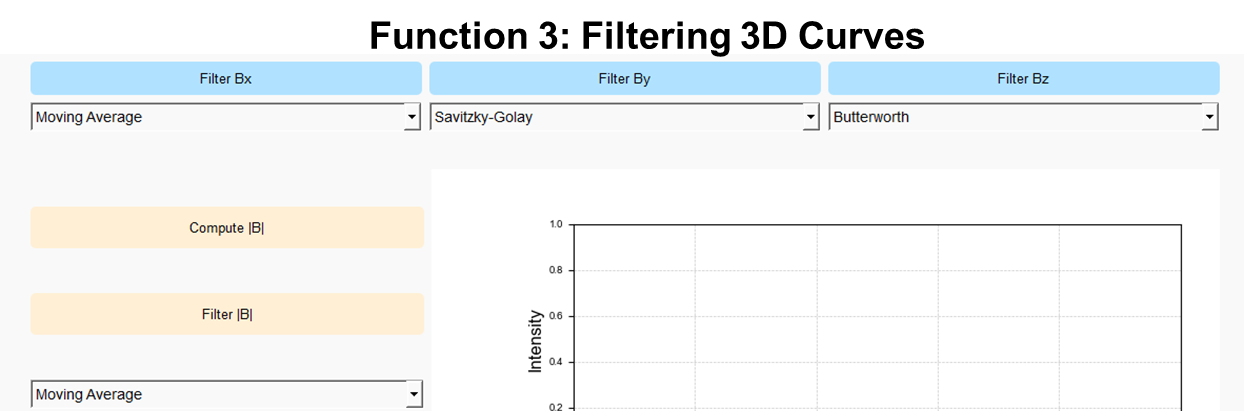
**

**Figure S13. Execution buttons for applying the selected filtering method to raw magnetic field data.**

**
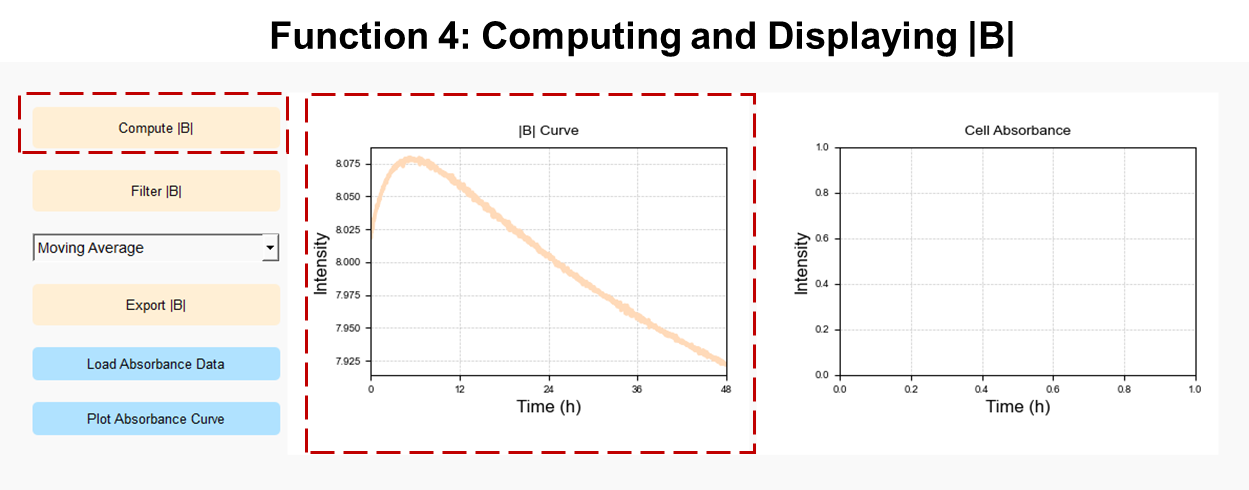
**

**Figure S14.** **Computation and visualization module for resultant magnetic field magnitude |B|.**

**
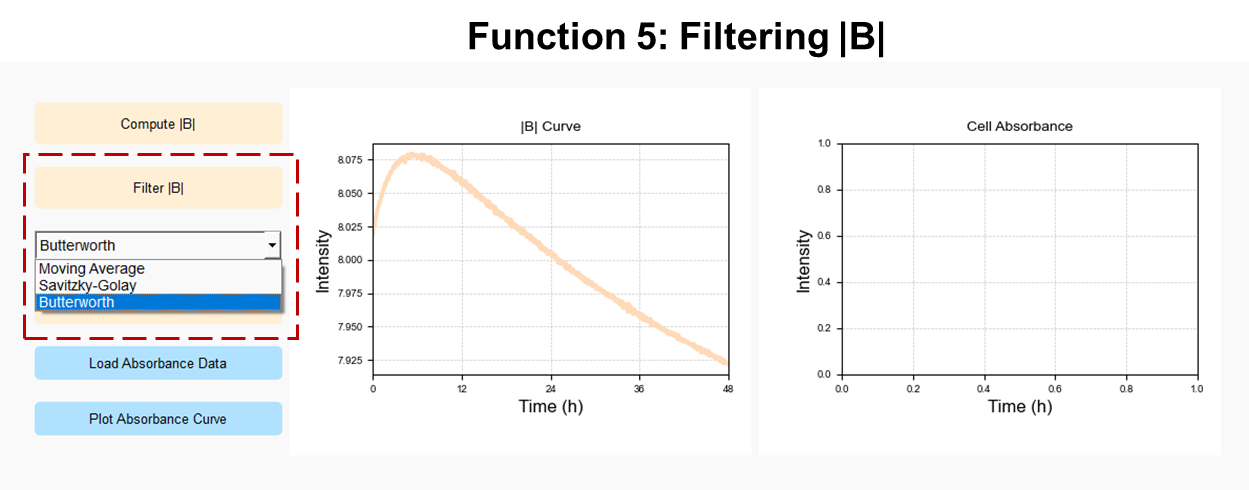
**

**Figure S15. Filtering options for post-processing the computed total magnetic field.**

**
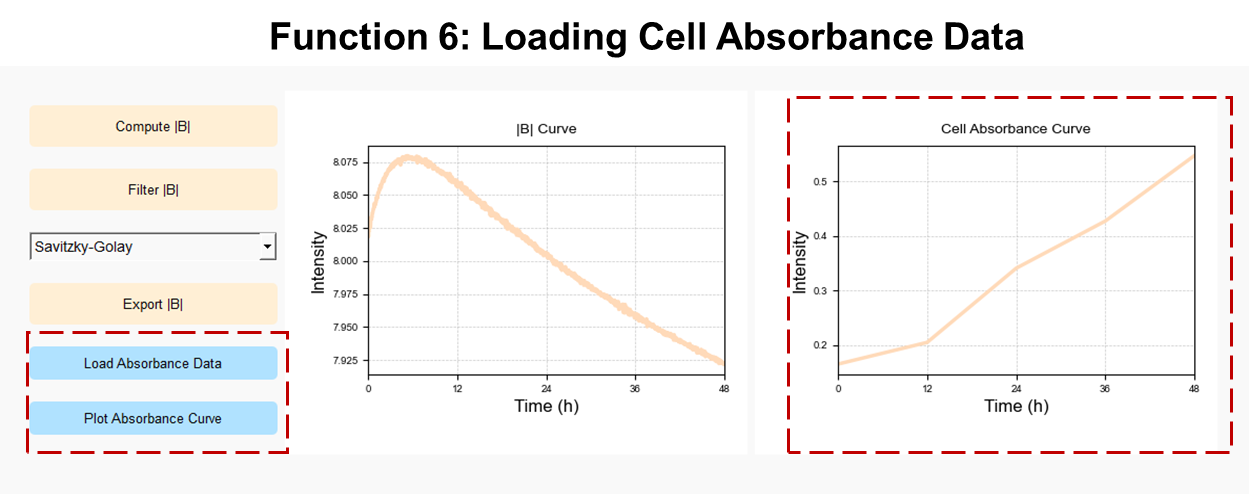
**

**Figure S16. Visualization of time-resolved cellular absorbance dynamics for proliferation analysis.**

To establish a closed-loop analytical workflow integrating sensing, visualization, and machine learning, we developed MagVizio—an object-oriented, modular software platform for real-time processing and interpretable rendering of magnetic field-based cellular dynamics **(Fig. S9-16).** Designed to maximize the utility of the MagMI system, MagVizio provides a graphical user interface (GUI) tailored to magneto-mechanical datasets, enabling real-time exploration and downstream integration with machine learning models.

As shown in **Fig. S9**, the software features a streamlined home interface, from which users can navigate to specialized processing modules. The functional overview panel **(Fig. S10)** displays modular subroutines that include 3D field rendering, multi-mode filtering, and signal-to-feature conversion. A cornerstone of the platform is dynamic 3D magnetic field visualization **(Fig. S11)**, which dynamically renders $B_{x},B_{y},B_{z}$ components over time, allowing users to visually assess cellular mechanical activity across spatial axes.

To improve signal interpretability and reduce noise from environmental artifacts, MagVizio incorporates three filtering strategies, Savitzky-Golay smoothing, moving average filtering, and Butterworth low-pass filtering **(Fig. S12)**. Each method can be selectively applied via execution buttons **(Fig. S13)**, enabling real-time preview and export of processed data. The software computes the resultant magnetic field magnitude |B| **(Fig. S14)**, which serves as the primary input for downstream machine learning pipelines.

In addition to raw component filtering, post-processing of the total magnetic field is supported via the same set of filters **(Fig. S15)**, ensuring analytical consistency across both vector and scalar field representations. Furthermore, MagVizio includes a cellular absorbance visualization module **(Fig. S16)**, which overlays predicted or experimentally derived cell proliferation curves for comparison, supporting temporal alignment with magnetic field changes and enabling kinetic correlation studies. This capability closes the loop between physical sensing and biological interpretation.

Taken together, MagVizio establishes an end-to-end framework that unifies physical signal acquisition, interactive visualization, and machine learning-ready data formatting. It empowers researchers to interactively explore subtle biomechanical signatures, apply customizable filtering strategies, and extract quantitative descriptors-all through a low-barrier, code-free interface. As such, MagVizio not only streamlines magneto-mechanical data interpretation but also accelerates adoption of AI-enhanced sensing in the broader life sciences community.
